# Activating a patient-relevant pathogenic variant of a genetically defined heart disease in a humanized pig model

**DOI:** 10.1038/s41684-026-01787-6

**Published:** 2026-09-11

**Authors:** Petra Runa-Vochozkova, Michaela Vaskovicova, Josep M. Cambra, Andrea Bähr, Eva-Maria Männer, Mayuko Kurome, Valeri Zakhartchenko, Tuna Güngör, Barbara Kessler, Heinke C. Heymer, Mara L. Corsten, Tarik Bozoglu, Vijayanand Rajendran, Solomon K. Yator, Tatjana Dorn, Jessica K. Hernandez, Ina M. Luksch, Magdalena Harakalova, Peter van Tintelen, Stefan Juhas, Jana Juhasova, Carolina G. Cabrera-Gómez, Susanne Zöls, Steffanie Senf, Francesca Stillitano, Joost P. G. Sluijter, Mathias Ritzmann, Zdenka Ellederova, Motlik Jan, Pieter A. Doevendans, David Drutovic, Pieter C. Glijnis, Eckhard Wolf, Christian Kupatt, Nikolai Klymiuk

**Affiliations:** 1 First Department of Medicine, Cardiology, Klinikum rechts der Isar, Technical University of Munich, School of Medicine and Health, Munich, Germany; 2 Center of Innovative Medical Models (CiMM), LMU Munich, Oberschleissheim, Germany; 3 Institute of Animal Physiology and Genetics, Czech Academy of Science, Libechov, Czech Republic; 4 Chair of Molecular Animal Breeding and Biotechnology, Gene Center and Department of Veterinary Sciences, LMU Munich, Munich, Germany; 5 Department of Cardiology, Laboratory of Experimental Cardiology, University Medical Center Utrecht, Utrecht, the Netherlands; 6 Regenerative Medicine Center Utrecht, Circulatory Health Research Center, Transplantation Center Utrecht, Utrecht University, Utrecht, the Netherlands; 7 Department of Genetics, University Medical Center Utrecht, Utrecht, the Netherlands; 8 Clinic for Swine, Center for Clinical Veterinary Medicine, LMU Munich, Oberschleissheim, Germany; 9 Stichting Genetische Hartspierziekte PLN, Wieringerwerf, the Netherlands

**Keywords:** Genetic engineering, Translational research

## Abstract

Complex diseases such as progressive cardiomyopathies are often insufficiently recapitulated in small animals or in vitro models. Large animal species such as pigs provide a valuable alternative, but constitutive genetic manipulation has not yet been applied to pigs in an effective manner, mainly due to biological and logistical limitations. Here we describe the generation of a humanized pig model for phospholamban-mediated cardiomyopathy and compare different methods for activating a pathogenic R14del mutation by Cre-mediated recombination. Both Cre treatment of pig primary cells before somatic cell nuclear transfer as well as microinjection of Cre-encoding mRNA into zygotes were similarly efficient in delivering piglets with an activated R14del mutation. Alternatively, administration of Cre-encoding adeno-associated virus into piglets was sufficient, albeit to a varying extent. Together, we describe a highly effective process to establish complex inducible genetic traits in pig and demonstrate that the lack of Cre-driver lines can be compensated by various interventions during reproduction or postnatally.

## Abstract (web summary)

The study describes the generation of a humanized pig model carrying an inducible phospholamban R14del mutation for cardiomyopathy modeling and demonstrates Cre-based activation strategies as efficient alternatives to germline Cre-driver lines in large animals.

## Main

An increasing number of cardiomyopathies, of either dilated, hypertrophic or arrhythmogenic phenotype, have been demonstrated to be caused by (likely) pathogenic genetic variants. Penetrance, severity and onset of the phenotype often vary in a patient population, and pathophysiological mechanisms involve secondary effects that are seemingly unrelated to the immediate genetic defect. Studying diseases such as arrhythmogenic cardiomyopathies in animal models also involves physiological and pathophysiological mechanisms that differ between model species and humans. For example, although frequently used for ischemic and hypertrophic cardiomyopathy studies, mice have a staggering heart rate and limited regulatory capacity of frequency that make it particularly difficult to directly translate results from mouse to human studies. Furthermore, due to its high resting frequency, the mouse heart is rarely tweaked into ventricular or atrial fibrillation, although high doses of caffeine and catecholamines may induce ventricular tachycardias.

By contrast, pigs offer an appealing alternative. Depending on their age and size, the heart of a pig can grow to a similar or even larger size as in humans, and notably share proportion, perfusion and valve mechanics. The pig heart has also been extensively studied through clinical grade electrophysiologic catheterization and high-resolution mapping^1^. Pigs do share the occurrence of ischemia-reperfusion arrhythmias and are additionally susceptible to ventricular tachycardias induced by exogenous beating cell transplants^2^. Finally, pigs can succumb to a sudden cardiac death phenotype, when (likely) pathogenic genetic variants are present, resulting in areas of low or no-amplitude action potentials in electrophysiologic maps. Of note, successful treatments preventing structural and intracellular deterioration during the unfolding of a genetic cardiomyopathy also result in electrophysiological stabilization in pigs^3^.

Since the implementation of highly potent CRISPR–Cas-based genome modification tools^4–7^, the expanding toolbox for genetic modification (GM) in pigs has increased their value as a model species for human diseases^8–10^. Examples of fast engineering include regulation of transgene expression^11,12^, establishing complex genetic traits such as the targeting of multiple sites^13,14^ and the (partial) humanization of disease-relevant genes^15^. In comparison, inducible GM (iGM), which involves switching genetic functions in certain cell types, defined developmental lineages or at desired time points by excision, insertion, inversion or exchange of defined genomic sites^16,17^, constitutes another level of complexity. In mice, iGM strategies have capitalized on recombinase enzymes that bind and tie defined recognition sites, with Cre enzymes recognizing lox segments of 34 bp being the most prominent example^18^ and FLTP-FRT or cphi31-attR systems serving as alternatives. Placing recombinases and their recognition sites as independent GM at distinct genomic localizations allows modular combinations or multiple independent modifications, such as genome-wide gene trapping^19^ or multicassette accumulation^20^. In pigs, GM lines for lox-based activation of reporter^21–23^ or oncogenes^24,25^ have their (theoretical) counterparts, in which Cre-recombinase expression is controlled by defined promoters, including *PTF1A*^26^, *AQP2*^27^, *VASA*^28^, *MX1*^29^ or *TYR*^30^. However, inherent biological constraints, such as longer reproduction cycles, larger size or maintenance costs, suggest that alternative strategies to combinatorial breeding are required to effectively implement iGM in pigs.

Here we present a pig model for a genetically inherited arrhythmogenic cardiomyopathy, caused by the frequent phospholamban (PLN) c.40_42delAGA; p.(R14del) pathogenic variant^31^, for which we combined comprehensive design with advanced GM strategies and Cre-mediated conditional activation of the pathogenic variant.

## Results

### Mining for PLN translational prospect

**Fig. 1:**
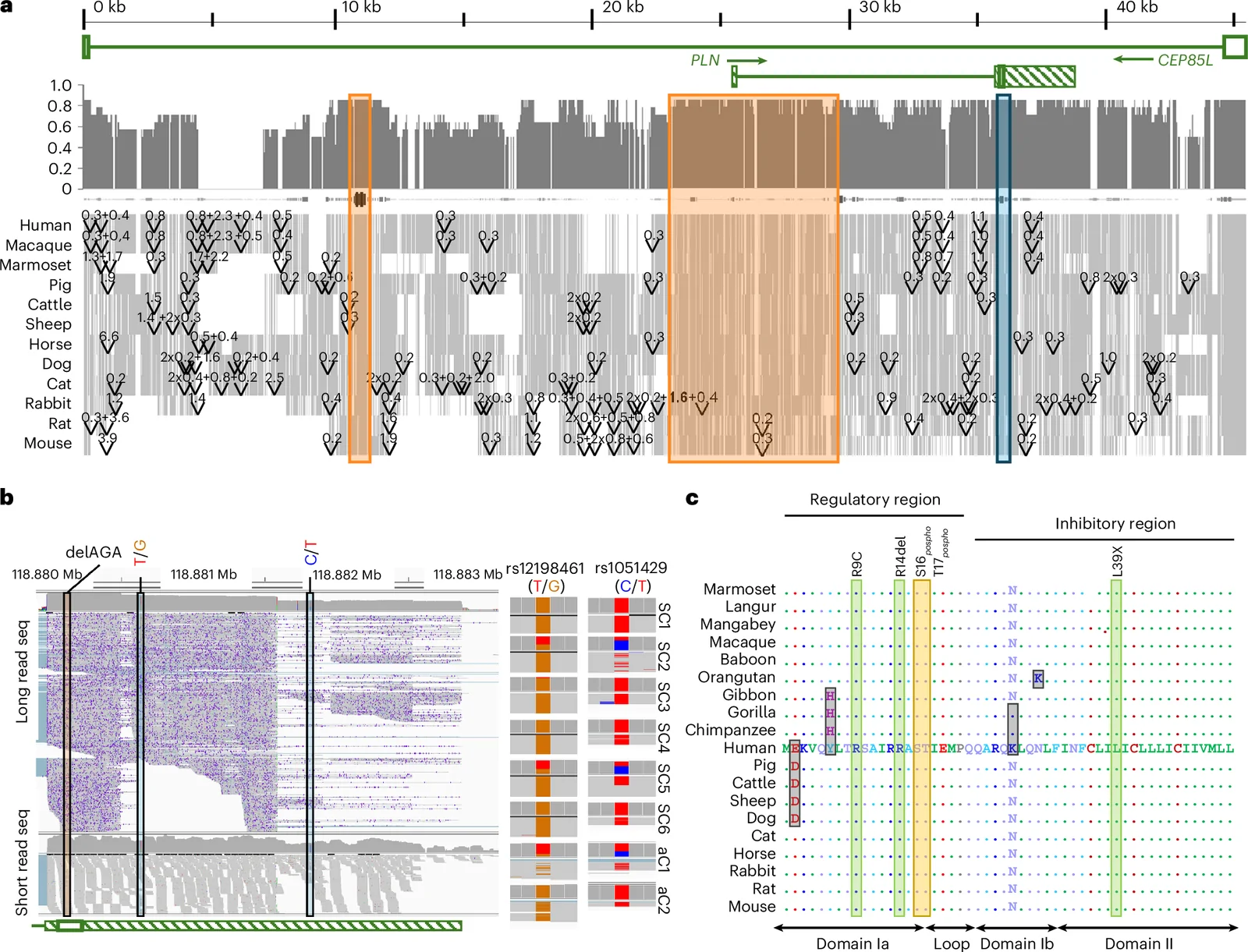
*PLN* cross-species analysis. **a**, Twelve mammalian *PLN* genomic loci within the adjacent exons of the surrounding *CEP85L* gene. From top to bottom, genomic organization, homology, regulatory regions and gene coverages are shown as in ref. ^15^. Repetitive elements occurring only in one of the major mammalian branches of primates, rodents, ungulates and carnivores were cleared from the alignment, but their positions and lengths (in kb) are indicated in the coverage. Sequence gaps are indicated in white for the respective species. A proposed enhancer region and the proximal promoter around *PLN* exon 1 are boxed in orange, while the *PLN* coding sequence is highlighted in blue. **b**, Representative deep sequencing, using long and short reads. The causative *PLN* c.40_42del mutation (delAGA, orange) and bystanding T/G and C/T SNV (blue) in the 3′-UTR are highlighted. Pattern of SNV in symptomatic (SC) and asymptomatic (aC) carriers. **c**, Mammalian PLN protein homology, using the human sequence as reference, while for other species only differing positions are indicated. Domains are represented according to ref. ^32^.

To assess the suitability of the pig (*Sus scrofa*) as a model species for PLN dysfunction, we explored the evolutionary relationship of PLN and its main interacting partners, the sarcoplasmic/endoplasmic reticulum calcium ATPase 1 (SERCA1; *ATP2A1*) and SERCA2 (*ATP2A2*) Ca^2+^ pumps across mammals. At the genomic level, multispecies alignments showed low similarity of the *PLN* genes among 12 species. Substantial homology was restricted to a potential enhancer element upstream of exon 1, the proximal promoter region surrounding exon 1 and the coding region in exon 2 (Fig. 1a). Besides a few conserved elements, such as the immediate poly-adenylation sites, the large 3′-untranslated region (UTR) revealed substantial divergence across species, including the integration of individual repetitive elements in several species.

Short- and long-read sequencing of transcripts in seven human PLN-R14del carriers strictly correlated the *PLN* c.40_42del variant (rs397516784) to the presumably benign rs12198461 and the rs1051429 single-nucleotide variants (SNVs) in the *PLN* 3′-UTR (Fig. 1b), supporting the hypothesis that the North-Western European *PLN*-R14del patients descend from a single founder event^32^.

At the protein level, the PLN membrane-spanning domain II and loop domain as well as the phosphorylation sites Ser16 (S16) and Thr17 (T17) were conserved among mammals (Fig. 1c).

**Fig. 2:**
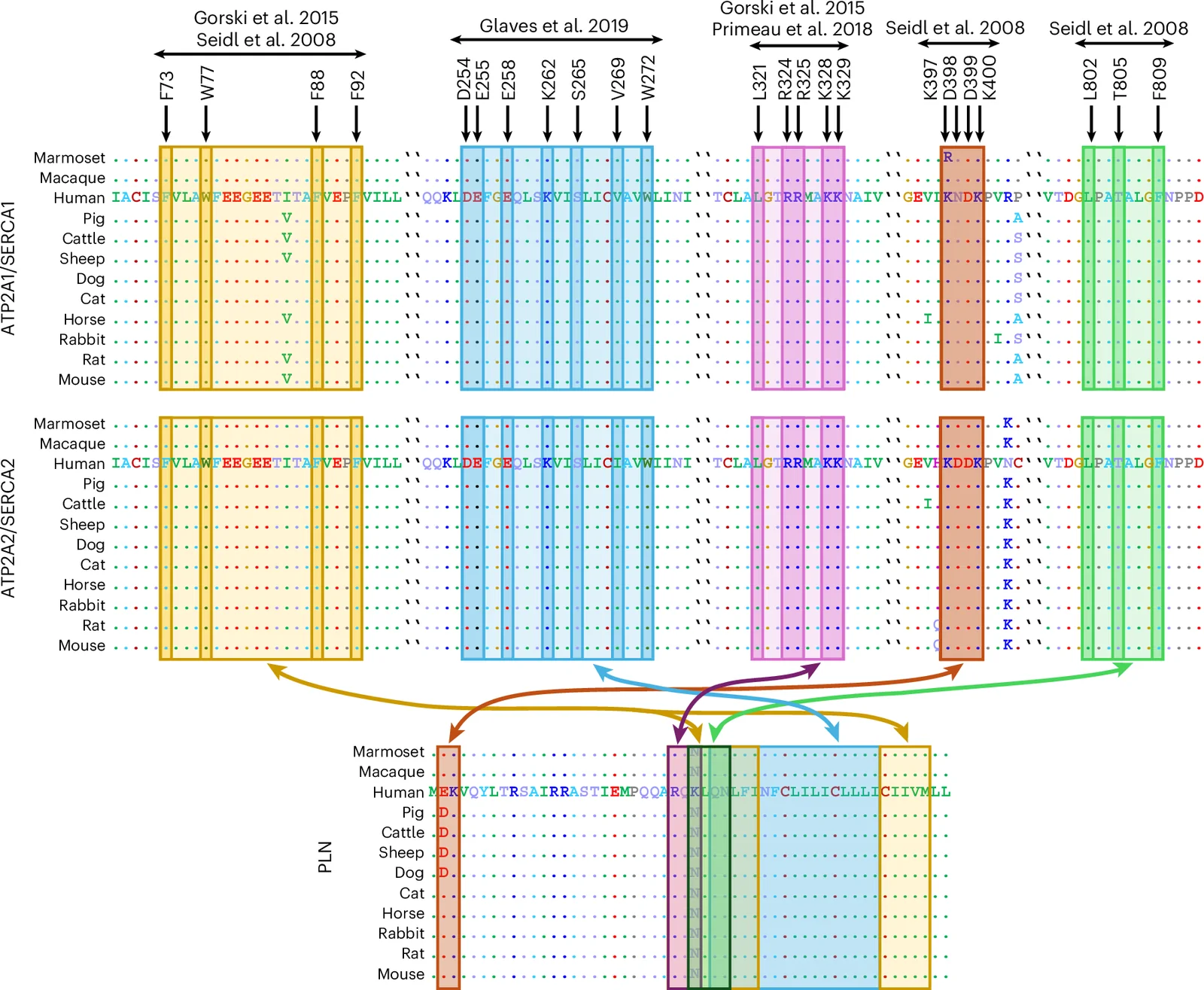
PLN–SERCA interactions. Domains and involved amino acids of SERCA1 (*ATP2A1* gene) and SERCA2 (*ATP2A2* gene) are highlighted and correlated to interacting segments in PLN by color coding and arrows, according to Seidel et al.^72^, Gorski et al.^73^, Glaves et al.^51^ and Primeau et al.^74^. The alignments are from the N to C terminus, with the PLN alignment shown in full length and the five SERCA domains separated by double prime symbols.

Variations in the regulatory and inhibitory regions of PLN included p.3D/E, p.7C/H (both domain Ia) and p.27K/N (domain Ib) and indicated species-specific properties regarding phosphorylation of PLN and interaction with SERCA. By contrast, the domains in the SERCA1 and SERCA2 proteins that interfere with PLN were identical between all examined mammals and differed only minimally between SERCA1 and SERCA2 (Fig. 2). Thus, we conclude that any mammalian PLN would interfere with pig SERCA proteins, but regulatory properties of pig and human PLN on SERCA activity may differ due to varying stability of mRNA, protein phosphorylation and affinity to SERCA.

### PLN modification strategy

**Fig. 3:**
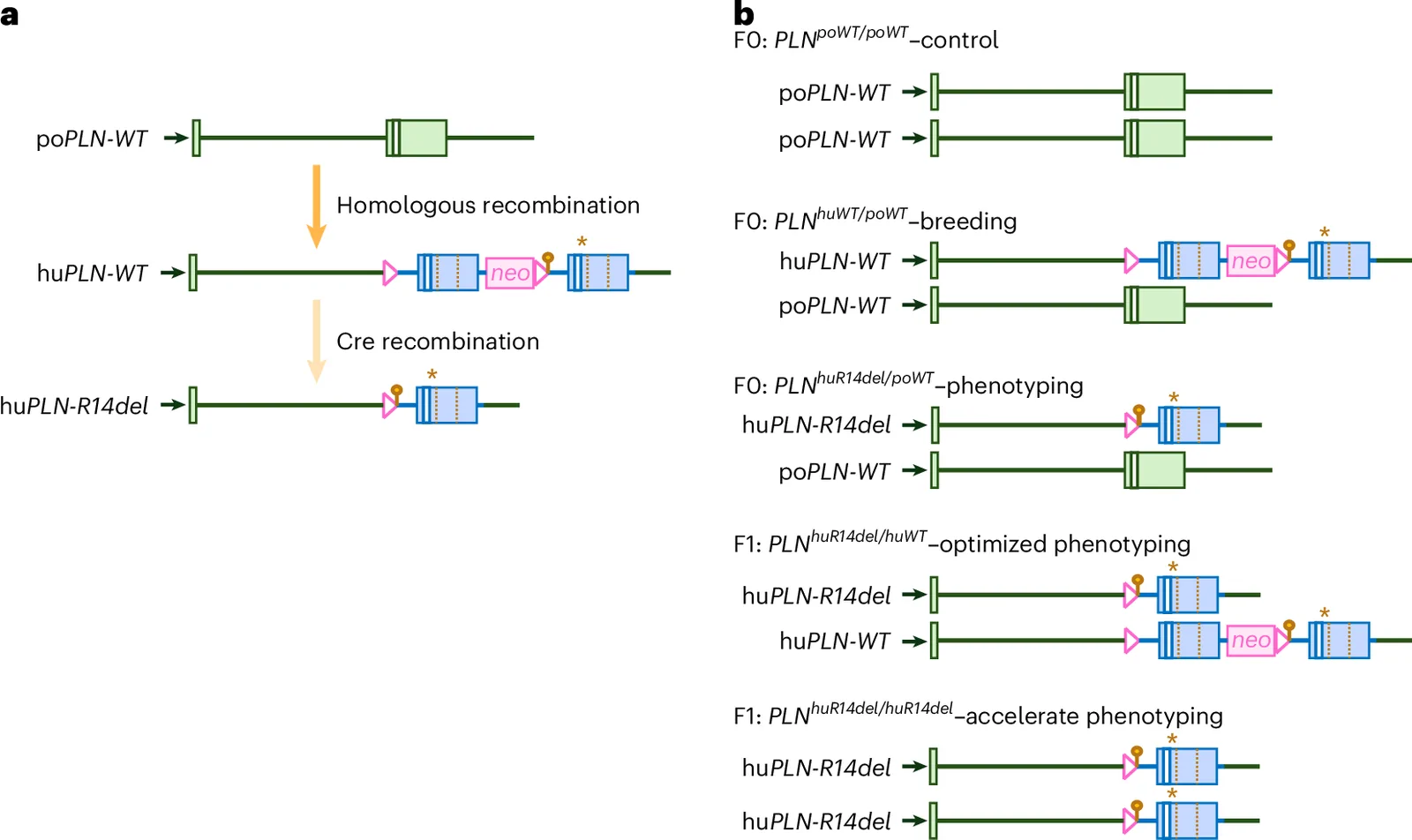
*PLN* transformation strategy and allelic constellations. **a**, The pig *PLN* locus (top) was first transformed into a modified allele that comprised a removable cassette with a hu*PLN-*WT exon 2 and a positive selection cassette as well as constitutively silent hu*PLN*-R14del cassette (middle). The hu*PLN*-R14del cassette was activated by Cre-lox mediated excision (bottom). Triangles, loxP sites; dotted lines, rs12198461 and rs1051429; asterisk, R14del modification; Pin, SNP variant discriminating hu*PLN*-WT and hu*PLN*-R14del cassettes. **b**, The iGM strategy facilitates genetic constellations for different purposes. In the founder generation, homozygous *PLN*^*poWT/poWT*^ serves as a control; heterozygous *PLN*^*huWT/poWT*^ constitutes healthy breeding animals; heterozygous *PLN*^*huR14del/poWT*^ gives first insight into the consequences of mutated *PLN* in pig cardiomyocytes. By combinatorial breeding, *PLN*^*huR14del/huWT*^ reconstitutes the allelic constellations of human patients in the pig heart. *PLN*^*huR14del/huR14del*^ is expected to lead to an accelerated phenotype, like the pathologies in homozygous mutant PLN mice^34,35^.

To mimic the action of human wild-type (WT) and defective PLN in a pig model, we proposed that the GM design must integrate (1) the causative mutation itself, (2) the entire human PLN protein constituents and (3) the main post-transcriptional regulatory properties of the extended 3′-UTR. The transition of PLN protein between mono- and pentameric stages raised questions about the compatibility of human and pig PLN and suggested (4) the combination of human intact (hu*PLN*-WT) and mutated (hu*PLN*-R14del) proteins. Finally, the dominant negative pathology demanded (5) a fallback strategy to prevent interference of disease expression with reproduction capacities. To efficiently integrate all requirements, we used an iGM strategy, using a double-cassette approach (Fig. 3a). In the initial configuration, the mutated PLN-R14del variant was silenced by an upstream element that served three purposes: establishing antibiotic resistance, providing a rescue strategy in case of deleterious R14del and allowing the complementation of hu*PLN*-R14del with huPLN-WT expression in a fully humanized hu*PLN*^*WT/R14del*^ locus after combinatorial breeding (Fig. 3b). Optionally, this approach can lead to the generation of a homozygous hu*PLN*^*R14del/R14del*^ variant, presumably leading to an accelerated phenotype^33^.

**Fig. 4:**
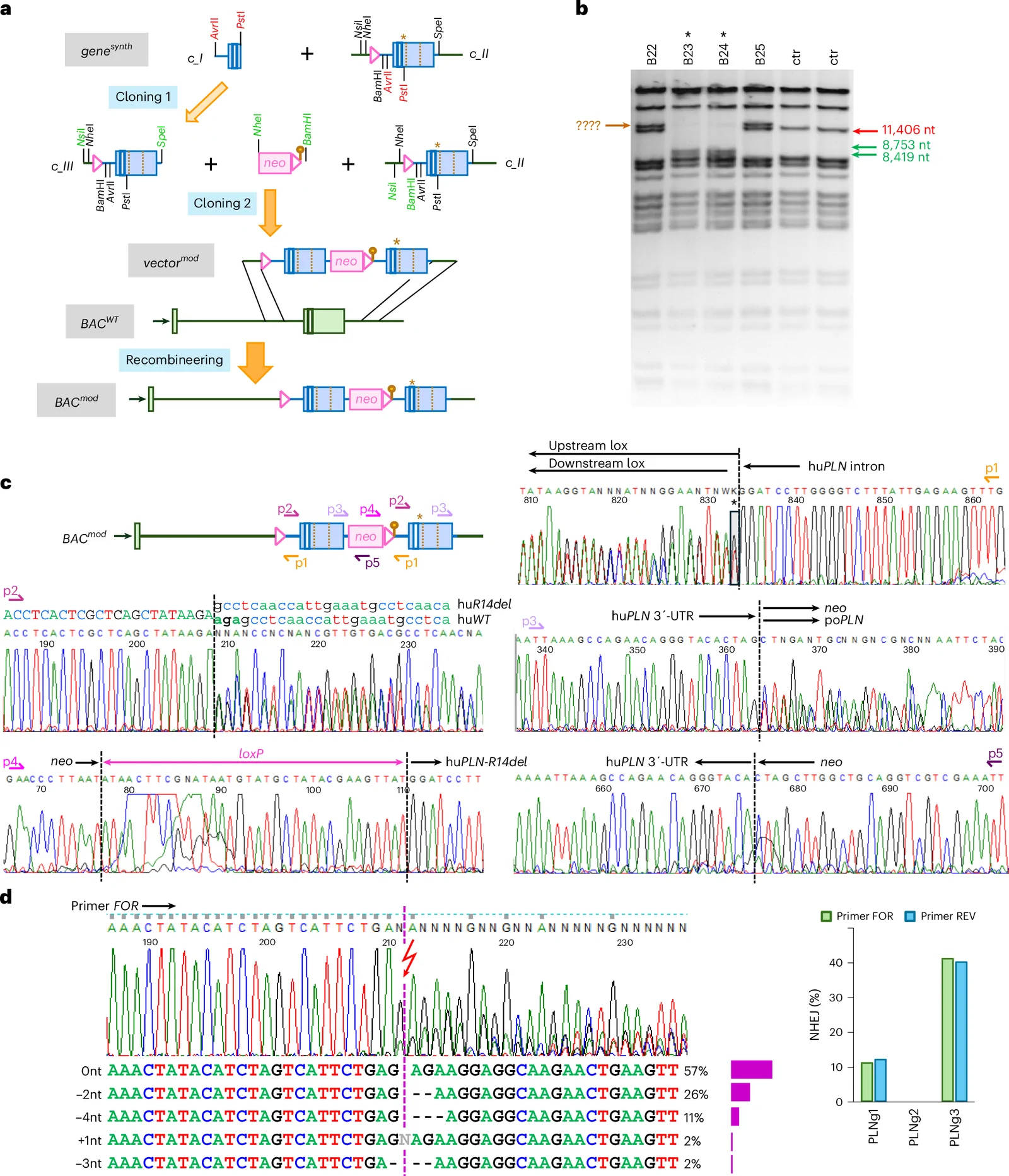
GM elements. **a**, Vector^mod^ was generated from two synthesized elements and a neo-expressing cassette in two-plasmid cloning steps and used to transform BAC CH242-318M5 (BAC^WT^) into a targeting vector (BAC^mod^). **b**, RE fingerprinting was used to confirm overall integrity and correct bacterial recombineering in BAC^mod^ clones, compared with original BAC (ctr). Exchange of an 11,406-nt fragment with 8,753-nt and 8,419-nt fragments, indicating correct insertion of vector^mod^, was observed in B23 and B24 (asterisks), while B22 and B25 had an aberrant RE pattern (questions marks). **c**, BAC sequencing was performed to verify the junction between GM key elements in BAC^mod^. Positions and orientations of primers are indicated. For better orientation, Sanger electropherograms from reversely orientated primers were transformed in silico. Junctions between key elements are marked. Due to primer binding at both hu*PLN*-WT and hu*PLN*-R14del elements in BAC^mod^, a mixed sequencing pattern is observed: for p1 (reverse, top right) caused by the ‘GG’ or ‘GGG’ segments (asterisk) at the junction between the hu*PLN* intron and the lox sites; for p2 (forward, middle left) caused by the presence or lack of the AGA triplet encoding R14del (bold); for p3 (forward, middle right) caused by the junction between the human 3′-UTR and the neo cassette or the segment downstream of the GM. The palindromic structure of the lox sites compromises sequencing quality (bottom left). **d**, gRNAs were tested for their efficacy to introduce NHEJ-mediated mutations in pig PKCs. Top left: representative Sanger electropherogram. Bottom left: calculated frequency of NHEJ variants by Synthego. Right: summary of NHEJ frequency with three gRNAs, sequenced in both orientations. Source data

Technically, the entire GM was first integrated into the *PLN* pig locus of pig primary kidney cells (PKCs) by homologous recombination (HR), while the activation of the PLN-R14del was subsequently achieved by Cre-lox mediated excision of the upstream cassette. Building on previous work, the HR protocol combined a modified bacterial artificial chromosome (BAC) vector^34,35^ carrying the desired GM within extended regions of homology, with CRISPR/Cas9 to induce double-strand breaks (DSBs) in the *PLN* locus and promote HR^36^. The modified BAC vector was created from a clone covering the pig *PLN* locus (CH242-318M5) using gene synthesis, sequential plasmid cloning and bacterial recombineering (Fig. 4a) and then validated by endpoint polymerase chain reaction (PCR) spanning homologous arms (Fig. 4c) and restriction enzyme (RE) fingerprinting^37^ (Fig. 4b). To induce DSBs in the *PLN* locus of pig PKC, three gRNAs were tested, revealing substantially distinct nonhomologous end-joining (NHEJ) mutation rates (PLNg1: 11–12%, PLNg2: 0% and PLNg3: 40–41%) (Fig. 4d).

### Humanizing the pig *PLN* locus

**Fig. 5:**
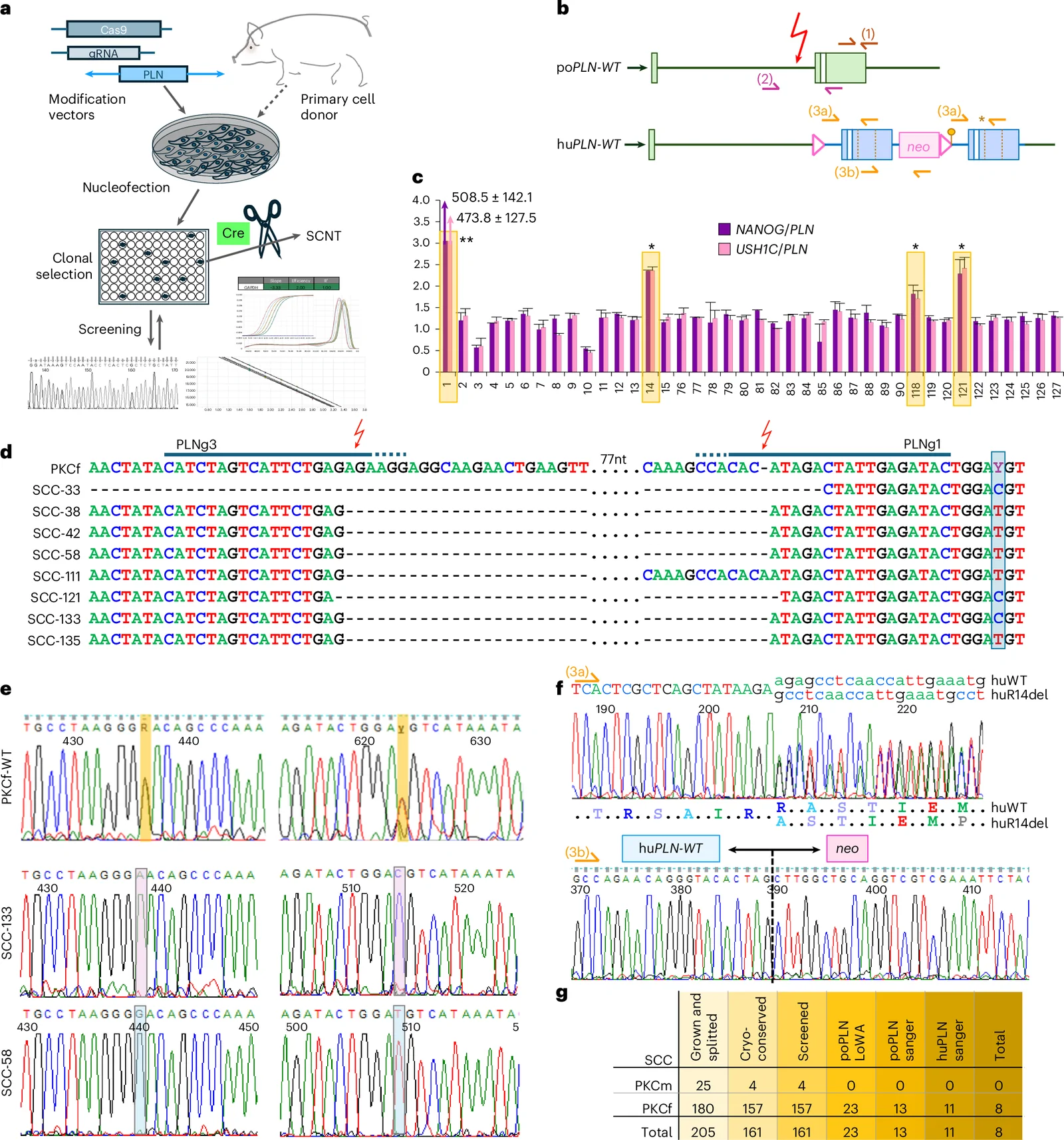
Generation and screening of genetically modified SCCs. **a**, PKC of a WT pig were nucleofected with GM components and seeded for clonal selection under neo treatment. After screening, verified SCCs were recovered and optionally treated with Cre-encoding mRNA before SCNT. **b**, Sequential screening of SCCs by (1) qPCR-based LoWA and Sanger sequencing of PCR amplicons spanning (2) the gRNA cutting site; (3a) the hu*PLN-*WT and hu*PLN*-R14del coding regions; and (3b) the junction between huPLN-WT and the neo cassette. **c**, Representative LoWA screening of a set of 40 SCCs, using qPCR_1 and *OCT4* and *NANOG* as reference genes for qPCR. Changes in *PLN* allele numbers are indicated by orange boxes. One asterisk indicates loss of 1 pig allele; two asterisks indicates loss of both pig alleles. **d**, NHEJ pattern of candidate SCCs was analyzed using PCR_2, following combined PLNg1 and PLNg3 use to stimulate HR. Positions of gRNA are indicated by the blue line, with the protospacer adjacent motif (PAM) represented as the dotted line. A naturally occurring SNV in the pig *PLN* locus identifies the haplotype of the remaining porcine *PLN* allele (blue box). **e**, Representative electropherograms across (G/A) and (C/T) SNV in PCR_2. **f**, Representative electropherograms of PCR_3a, confirming the presence of the huPLN-WT and huPLN-R14del coding regions (top) and of PCR 3b, confirming the correct transition from the huPLN-WT sequence to the neo cassette (bottom). **g**, Summary of the sequential SCC screening, confirming that eight clones fulfill all selection criteria.

**Fig. 6:**
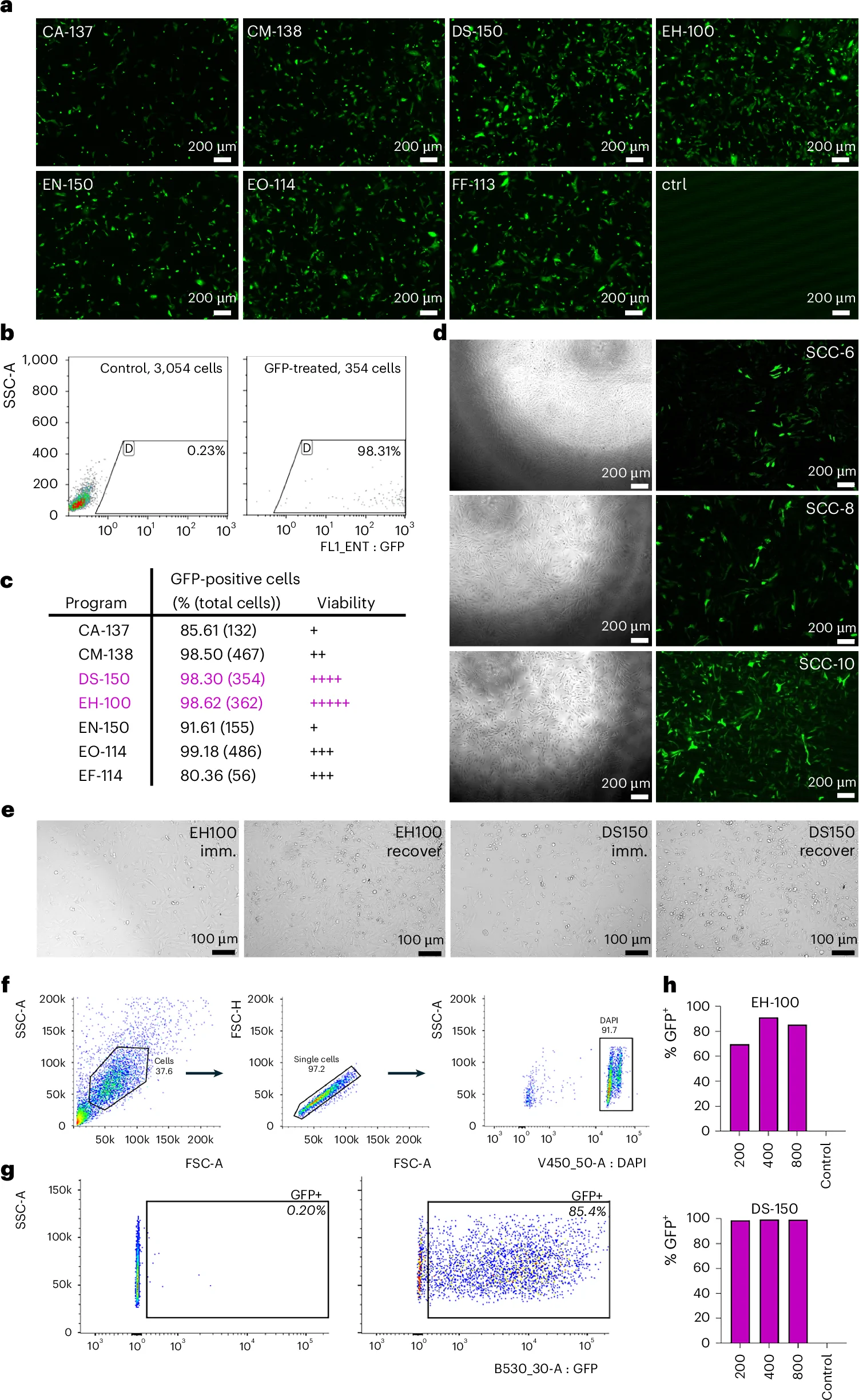
Activation of huPLN-R14del by renucleofection of SCCs. **a**, To optimize the nucleofection of small cell numbers, 10^4^ PKCs were treated with a GFP-encoding plasmid, using different Lonza 4D programs. **b**, Representative FC-based quantification of control and GFP-treated PKC. **c**, Nucleofection efficacy and cell viability using the Lonza 4D nucleofector, with best-performing programs highlighted. **d**, Representative brightfield and fluorescent microscopy images of selected SCCs after nucleofection with GFP plasmid. SCCs were unmodified clones from the PLN-targeting experiment (Fig. 5c). **e**, Brightfield microscopy of SCCs that were nucleofected and then seeded immediately (imm.) or after 10 min recovery in medium. **f**, Gating strategy for SCCs after 10 min recovery. **g**, Quantifying PKC after optimized nucleofection. **h**, Dose-dependent nucleofection with optimized protocol and best EH-100 and DS-150 programs.

According to established protocols (Fig. 5a), PKC of 2 distinct WT pigs were nucleofected with a linearized BAC vector and plasmids expressing Cas9 and guide RNAs (gRNAs). After clonal selection and passaging, 161 single-cell clones (SCCs) were cryo-conserved and screened for HR in a sequential protocol (Fig. 5b). Quantitative PCR (qPCR)-based loss-of-wild-type allele (LoWA) detected 100–6,000 genomic copies/µl in the DNA preparations of SCCs, excluding 138 clones from further evaluation (Fig. 5c). One of the 23 SCC candidates did not retain a pig *PLN* allele, suggesting bi-allelic modification. Sanger sequencing of PCR amplicons spanning the CRISPR–gRNA binding site leveraged the discriminating capacity of NHEJ-mediated mutations and two naturally occurring SNV in the pig *PLN* locus, confirming that 13 SCCs retained a single pig *PLN* allele (Fig. 5d,e). Most of these clones acquired an intronic gap of 111 nt that exactly matched the cutting sites of the gRNA, whereas an alternative NHEJ pattern was observed only occasionally. After final verification of the presence of the huPLN-WT, huPLN-R14del and neo-coding regions, a total of eight candidate clones were prepared for somatic cell nuclear transfer (SCNT) (Fig. 5f,g). To activate huPLN-R14del variants already in the founder generation (F0), some of the SCCs were nucleofected with Cre-encoding plasmid or lipofected with Cre-mRNA (Fig. 6).

**Fig. 7:**
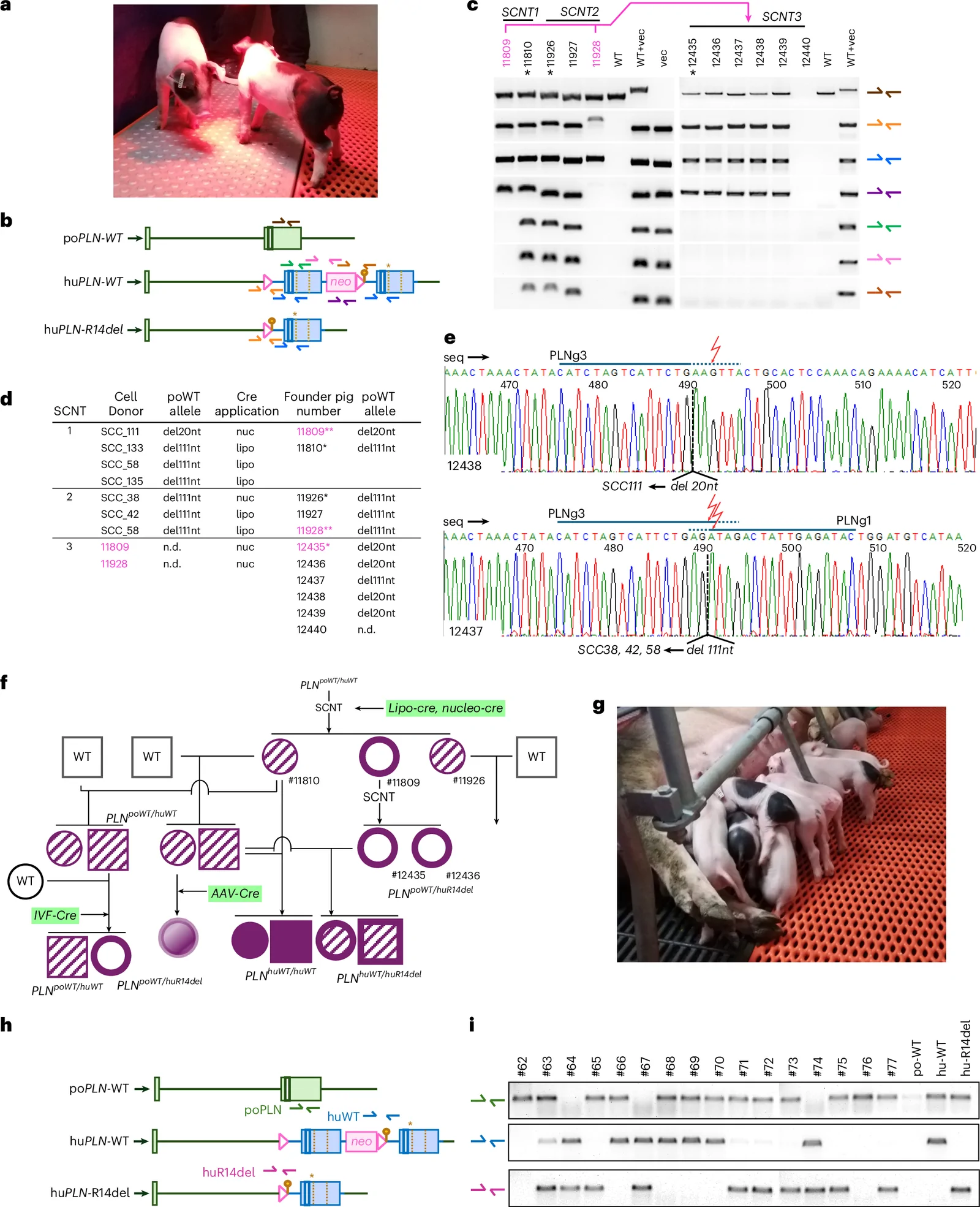
Generating and propagating *PLN* mutant pigs. **a**, Photos of SCNT-derived founder animals. **b**,**c**, The animals were screened by endpoint PCR assays (arrows indicated in schematic in **b**) to determine hu*PLN*-WT and hu*PLN*-R14del constellations (**c**). Color coding of PCR assays matches in **b** and **c**. Animal numbers in pink correspond to isolated PKC that were used in recloning. Asterisks: animals used to establish breeding colonies. **d**,**e**, NHEJ pattern (according to Fig. 5d) in verified SCCs and in SCNT-derived founder animals are described (**d**), after being identified by Sanger sequencing (**e**). *Breeding animals; **PKCs isolated for recloning. **f**, Breeding program to propagate PLN herd from #11809, #11810 and #11926 founder animals. Black: WT; shaded: heterozygous hu*PLN*-WT; monochrome: homozygous huP*LN*-WT; bold: hu*PLN*-R14del. Stage and method of Cre application are highlighted in green. **g**, F1 offspring from a mating of the #11820 female with a WT boar. **h**, Endpoint PCR to genotype po*PLN*-WT, hu*PLN*-WT and hu*PLN*-R14del in the breeding population. **i**, Representative genotyping of a litter derived from #11810 (hu*PLN*-WT) × #11809 (hu*PLN*-R14del) matings. Color coding of primers as in **h**. ND, not determined. Source data

**Fig. 8:**
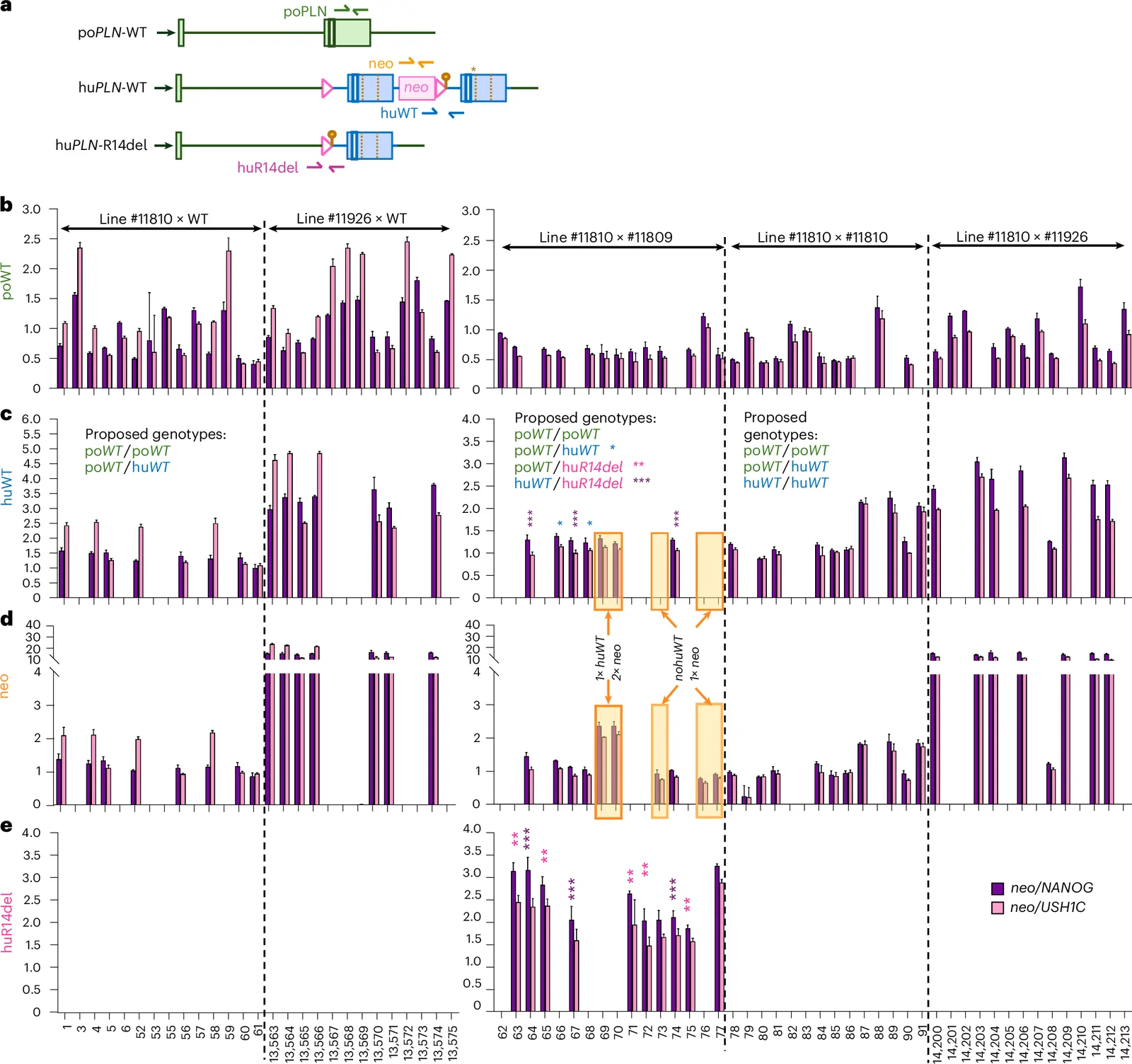
Quantitative inheritance of genetic elements during breeding. **a**, qPCR assays (color-coded arrows) were used to quantify the po*PLN*-WT, hu*PLN*-WT, neo and hu*PLN*-R14del elements in pigs from the breeding program (Fig. 7f). **b**–**e**, Relative copy numbers po*PLN*-WT (**b**), hu*PLN*-WT (**c**), neo (**d**) and hu*PLN*-R14del (**e**) were determined in representative litters from outbreeding (left) and inbreeding (right), using *NANOG* and *USH1C* as genomic reference loci. Parental genotypes are indicated for each litter. Litters are separated by dotted lines. For neo (**d**), the *y* axis was split to document the high number of copies (>20) in the 11926 line. For relevant matings, the expected genetic constellations are indicated, and for the #11810 × #11809 litter, offspring with expected pattern are marked by asterisks and piglets with aberrant patterns are boxed.

Four SCNT experiments were conducted, using pooled SCC preparations, according to previous work^38^, which resulted in three viable and two stillborn piglets in two litters (Fig. 7a). Genotyping by endpoint PCR (Fig. 7b,c) revealed that three of them carried the complete GM, whereas two lacked the hu*PLN*-WT cassette, indicating Cre-mediated excision. Sanger sequencing of PCR products spanning the modification segment confirmed the quantity and identity of hu*PLN*-WT and hu*PLN*-R14del genetic elements and the correct transition between them (Supplementary Fig. 1). Another SCNT experiment, using PKC of two deceased founder animals with excised hu*PLN*-WT cassettes, delivered five recloned offspring. A characteristic NHEJ-mediated gap of 20 nt on the porcine (po) *PLN* allele associated four of them to piglet #11809 and SCC_111. The origin of the other animal remained unclear due to the consistent deletion of 111nt at the DSB in the majority of SCCs used in SCNT (Fig. 7d,e). Animals #11810, #11926 and animal #12435—a viable reclone of #11809—were raised and propagated by a breeding scheme that combines outbreeding, for increased fertility and fitness, with inbreeding to achieve a hu*PLN*^*WT/R14del*^ genetic constellation at the *PLN* locus (Fig. 7f–i). Quantitative analysis of the po*PLN*-WT, hu*PLN*-WT, neo and hu*PLN*-R14del genetic elements (Fig. 8a) identified some aberrant genetic constellations. In line #11926, the appearance of mono- and bi-allelic po*PLN*-WT copies during breeding (Fig. 8b) confirmed the modification of the *PLN* locus. The abundance of several (3–4) hu*PLN*-WT and multiple (>20) copies of neo, however, suggested that neo resistance did not result from HR of the BAC vector within the *PLN* locus but by the integration of concatemeric vector fragments (Fig. 8c,d), similar to what has been reported with microinjection in mouse zygotes^39^. Quantitative validation in line #11809 confirmed that the inheritable hu*PLN*-R14del genotype (Fig. 8e) segregated from a fragmented neo-positive vector element during breeding, according to the Mendelian rules of inheritance. As a result, line #11926 was removed from the breeding schedule, whereas line #11810 served as a founder for the hu*PLN*-WT population; line #11809 founded the hu*PLN*-R14del genotype after outbreeding of the neo segment.

### Activation of hu*PLN*-R14del by mRNA microinjection

**Fig. 9:**
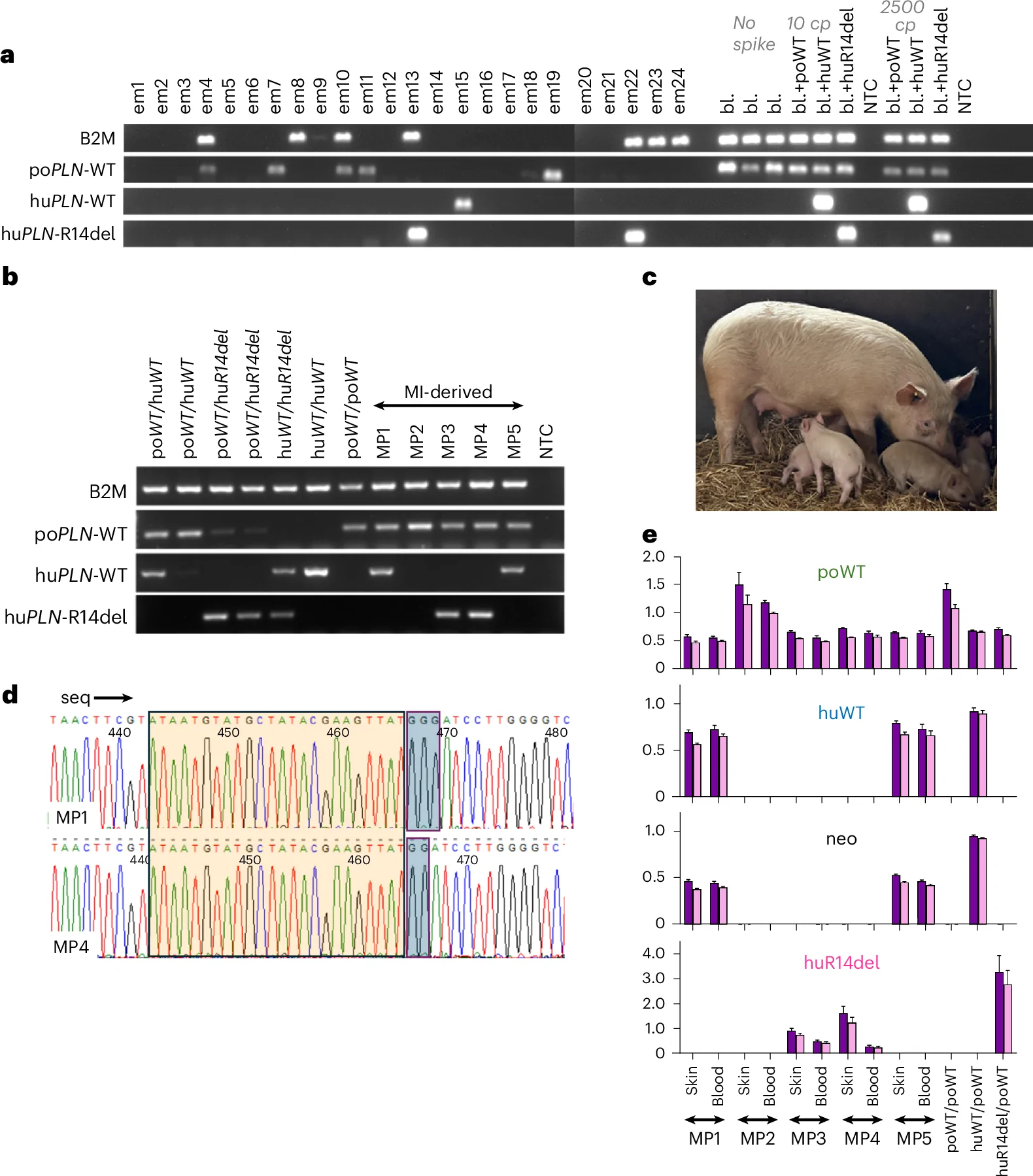
Activating hu*PLN*-R14del genotype by zygote injection. **a**, Nested-PCR screening of embryos em1–em24, derived from hu*PLN*-WT × WT matings after microinjection with Cre mRNA into zygotes and in vitro cultivation for 4–5 days. Blastocysts (bl.) derived from parthenogenesis were spiked with varying amounts of gDNA from po*PLN*-WT, hu*PLN*-WT and hu*PLN*-R14del animals to constitute control samples of distinct genotypes. Amplicons from *B2M* and po*PLN* loci represent positive controls, whereas PCR for hu*PLN*-WT and hu*PLN*-R14del alleles were used to discriminate the status of the GM. **b**, Genotyping of a microinjection (MI)-derived litter, using gDNA isolated from skin. Piglets from the breeding herd (Fig. 7f) served as controls. **c**, Photos of a MI-derived litter. **d**, Representative electropherograms of MI-derived piglets spanning the upstream loxP site (orange box); these were used to discriminate between hu*PLN*-WT and hu*PLN*-R14del genotypes based on the characteristic ‘GGG’ and ‘GG’ motifs (blue boxes), as shown in Fig. 4c. **e**, Quantification of relative copy numbers of the po*PLN*-WT, hu*PLN*-WT, neo and hu*PLN*-R14del elements in skin and blood samples. Controls as in **b**. Source data

As an alternative to segregate the aberrant neo cassette from *PLN*-R14del line #11809 by breeding, we aimed at Cre-mediated excision of the floxed hu*PLN*-R14del/neo cassette in the pure *PLN*-WT line #11810. Sperm of a hu*PLN*-WT F1 boar was used to inseminate synchronized WT sows of the Libechov minipig breed. The next day, sows were slaughtered, and presumptive zygotes were flushed from the oviduct, cultivated in improved defined medium and injected with highly pure mRNA coding a codon-optimized variant of Cre recombinase^40^. Developmental capacity and recombination efficiency were first tested in vitro. After cultivation for 4–5 days, 58.33% of microinjection-derived embryos reached morula or blastocyst stages, indicating appropriate developmental potential. Although genotyping of in vitro cultured embryos was not fully conclusive, at least 2 of 23 embryos showed the desired hu*PLN*-R14del constellation, thereby confirming correct excision of huPLN-WT after zygote injection of Cre mRNA (Fig. 9a).

Next, injected zygotes were cultivated for one day to 2–4-cell stages, and then approximately 30 embryos were transferred to synchronized gilts. One pregnancy was established from three embryo transfers and delivered five viable offspring of the proposed *PLN*^*poWT/poWT*^, *PLN*^*poWT/huWT*^ or *PLN*^*poWT/huR14del*^ genotypes (Fig. 9b,c). To assess mosaicism in microinjection-derived piglets, PCR products spanning the first loxP site were sequenced, revealing that piglets unambiguously retained the ‘GGG’ signature, indicative of hu*PLN*-WT, or the ‘GG’ signature, confirming hu*PLN*-R14del. (Fig. 9d). In parallel, qPCR-based copy number analysis of genomic DNA (gDNA) from skin (ectoderm-derived) and blood (mesoderm-derived) showed concordant genotypes between tissues: MP1 and MP5 displayed a hu*PLN*-WT pattern, whereas MP3 and MP4 showed hu*PLN*-R14del, with no evidence of residual hu*PLN*-WT or neo sequences, indicating absence of mosaicism (Fig. 9e).

### Postnatal activation of hu*PLN*-R14del genotype

**Fig. 10:**
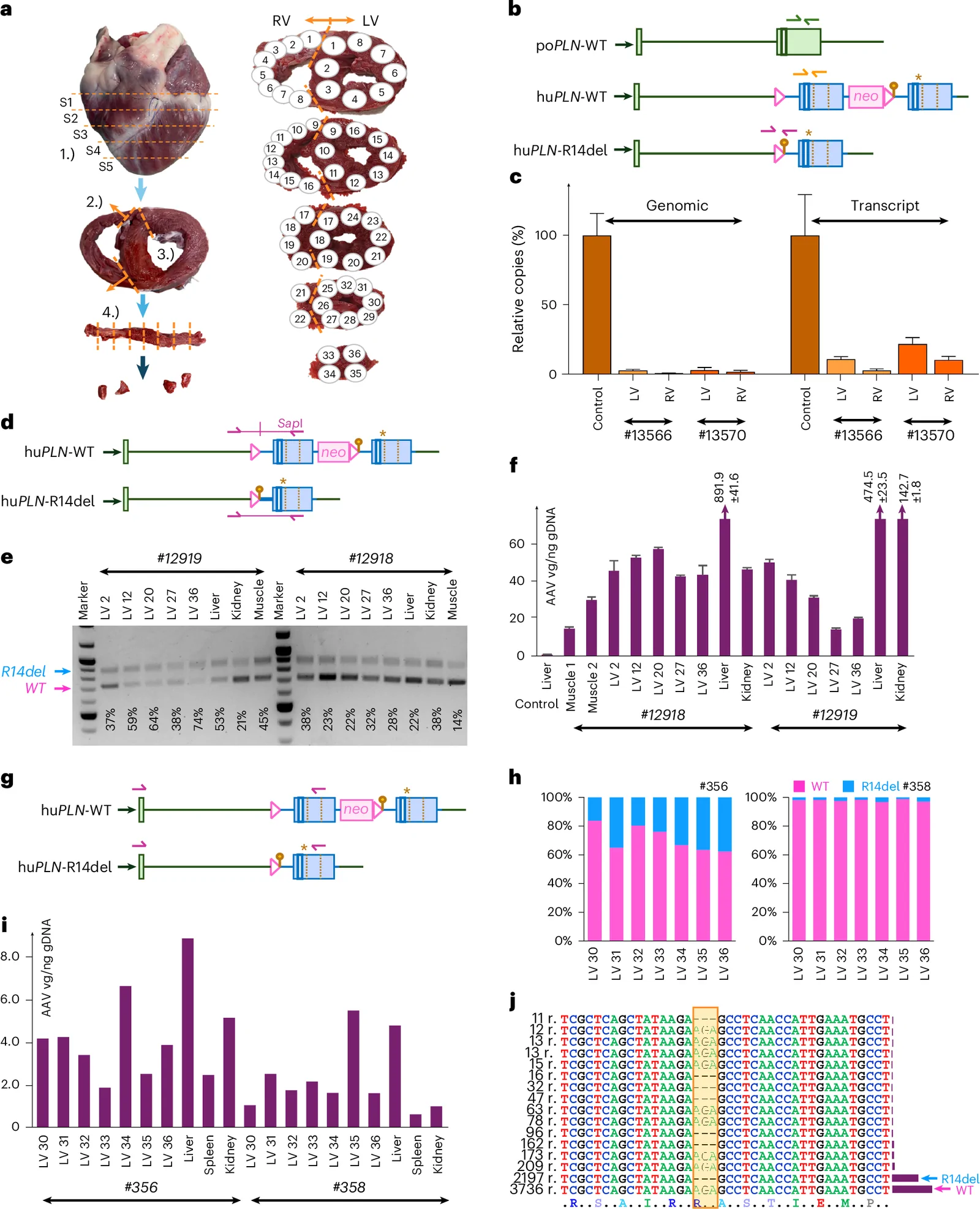
Postnatal activation of the hu*PLN*-R14del genotype by AAV2/9-mediated Cre delivery. **a**, Hearts were collected 3–30 weeks after injection to obtain spatially assigned tissue pieces from LV and RV in four steps: (1) division into five segments; (2) separation of the RV; (3) opening of the LV; and (4) segmentation of the linearized heart fragment into two to eight tissue pieces. In total, 36 LV and 22 RV samples were mapped across the heart (right). **b**,**c**, Specific qPCR assays were used to detect genomic (Fig. 8a) and transcript levels (**b**) of hu*PLN*-WT and hu*PLN*-R14del in the heterozygous *PLN*^*poWT/huWT*^ animals after injection with AAV without coating (**c**). Genomic copy numbers in treated animals (#13566: 6.0 × 10^15^ vp, #13570: 8.4 × 10^15^vp) were normalized to *NANOG* and *OCT4*, whereas transcript copy numbers were normalized to *ACTB*, *PPIA*, *TBP* and *GAPDH*. Values were subsequently normalized to hu*PLN*-R14del genomic and transcript levels in two *PLN*^poWT/huR14del^ pigs. **d**, *Sap*I-mediated RE fragment length polymorphism was used to discriminate between hu*PLN*-WT and hu*PLN*-R14del. **e**, Densitometric quantification of PCR products following SapI digestion after injecting *PLN*^*poWT/huWT*^ pigs #12918 and #12919 with 1.0 × 10^15^vp AAV-Cre + 180 µg G2-cys. Percentages indicate the densitometric proportion of the R14del band. **f**, Vector genome copy numbers in the respective heart samples and off-target tissue from animals #12918 and #12919. **g**,**h**, Next-generation sequencing with specific primers (**g**) was used to quantify hu*PLN*-R14del transcripts in heart samples from homozygous *PLN*^huWT/huWT^ pigs after treatment with coated AAV (1.0 × 10^16^vp + 180 µg G2-myoP8) (**h**). **i**, Virus copy numbers in the heart samples and off-target organs. **j**, Representative profiling data by next-generation sequencing of the LV36 sample of pig #356 with the AGA codon of R14 boxed. Besides dominant WT (49.23%) and R14del (28.95%) reads (arrows), minor variants were characterized by point mutations outside of the selected region and presumably represent PCR artifacts. Source data

To account for the possibility that homozygous *PLN*^*huR14del/R14del*^ genotypes might experience developmental setbacks during breeding, we sought to activate the hu*PLN*-R14del cassette in viable animals after birth. At an age of 4–8 weeks, heterozygous *PLN*^*poWT/huWT*^ or homozygous *PLN*^*huWT/huWT*^ F1 piglets were injected intravenously with 1.0 × 10^15^–1.0 × 10^16^ vp of Cre-encoding adeno-associated virus (AAV)2/9, with or without poly(amidoamine) (PAMAM) coating or with improved cardiomyocyte transduction capabilities. After termination, heart tissue was systematically sampled from the left ventricle (LV) and right ventricle (RV) (Fig. 10a) and examined for virus abundance and activation of the hu*PLN*-R14del cassette. In a pair of heterozygous *PLN*^*poWT/huWT*^ animals, injected with AAV without coating and maintained for 21 weeks post-infection, Cre-activated hu*PLN*-R14del cassettes ranged between 0.6% and 3.8% in LV and 0.4% and 2.1% in RV samples at the genomic level, whereas hu*PLN*-R14del transcripts reached 9.7–27.9% for LV and 0.9–13.4% for RV (Fig. 10b,c). In another pair of *PLN*^*poWT/huWT*^ animals, coating AAV with G2-cys PAMAM showed improved activation of hu*PLN*-R14del and substantial amounts of virus genomes in defined heart samples, 3 weeks after virus administration. (Fig. 10d–f). Coating with G2-myoP8 PAMAM resulted in varying efficacy in homozygous *PLN*^*huWT/huWT*^ pigs, with hu*PLN*-R14del accounting for 16–37% of *PLN* transcripts in one pig, but only 2% in the other pig, albeit the amount of virus genomes was comparable in the respective samples (Fig. 10g–j).

### Transcriptional configurations at modified *PLN* loci

**Fig. 11:**
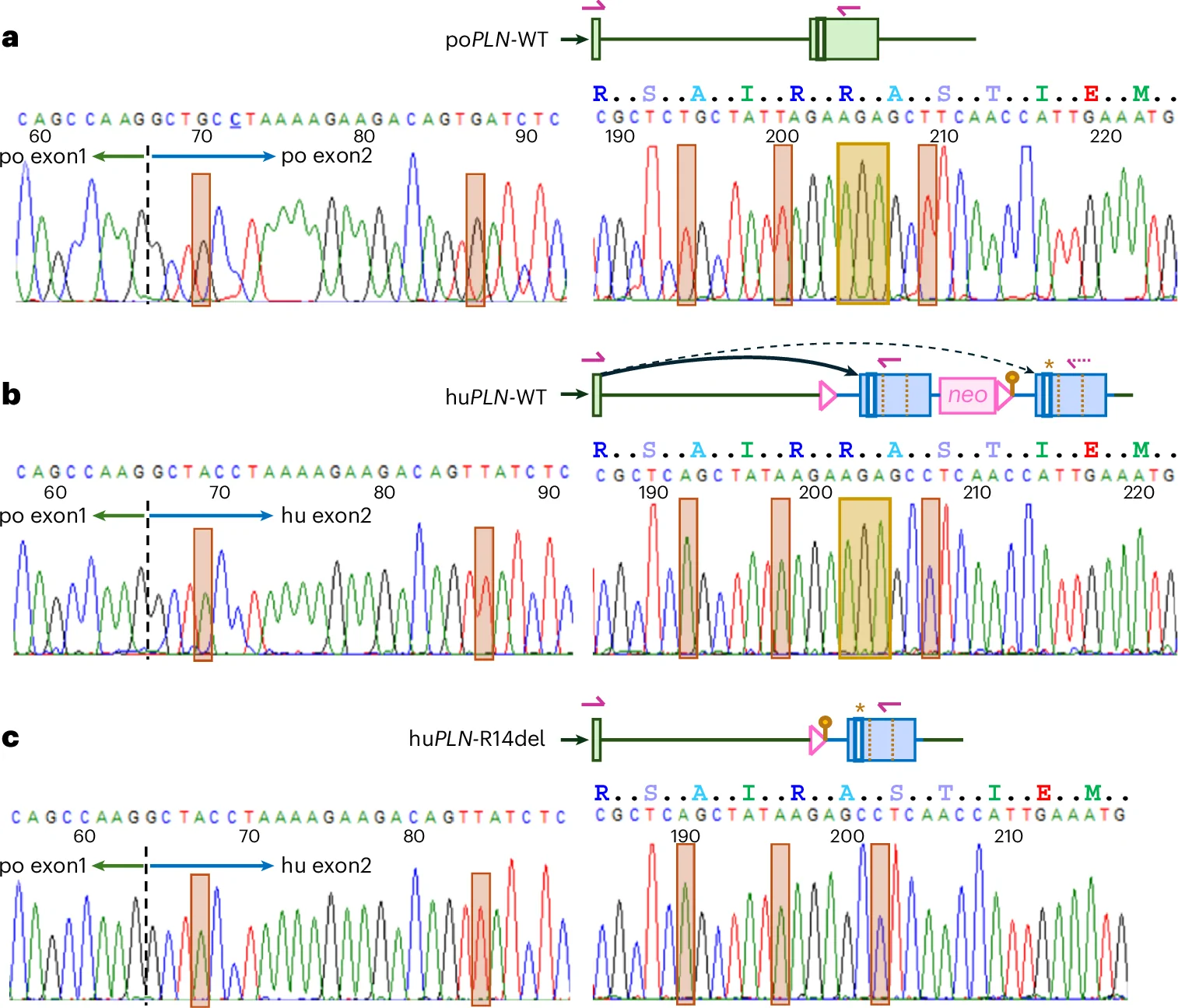
Transcript variants from modified *PLN* loci. **a**–**c**, RT–PCR span from po*PLN* exon 1 to the respective po*PLN*-WT (**a**), hu*PLN*-WT (**b**) and hu*PLN*-R14del (**c**) downstream exons (pink arrows). Exon–exon boundaries are consistent between pig PLN and humanized PLN variants (left, dotted lines). The amino acid sequences correspond to their codon pattern (right). AGA-codons in po*PLN*-WT and hu*PLN*-WT alleles are boxed in gold. SNV discriminating pig and human *PLN* exon 2 are boxed in brown. In **b**, the proposed dominant splicing to the hu*PLN*-WT and the aberrant splicing to the hu*PLN*-R14del cassettes are indicated (solid and dotted black arrows).

Sanger sequencing was performed on reverse-transcription PCR (RT–PCR) amplicons that span the consistent po*PLN* exon 1 and the different po*PLN*-WT, hu*PLN*-WT and hu*PLN*-R14del downstream exons to verify correct expression of the respective allele. In *PLN*^*poWT/poWT*^ control pigs, exon boundaries and coding sequence (Fig. 11a) were in line with GenBank entries, confirming the presumed conserved splice donor and splice acceptor sites in the multispecies alignment (Fig. 1a). In *PLN*^*poWT/huWT*^ pigs, RT–PCR specifically amplified humanized transcripts and verified the predominant splicing of po*PLN*-WT exon 1 to hu*PLN*-WT exon 2, thereby confirming that splice acceptor sites remained functional when transferred across species (Fig. 11b). Low-frequency (<5%) alternative splicing occurred at the downstream hu*PLN*-R14del exon, consistent with effective silencing of the mutated cassette. In *PLN*^*poWT/huR14del*^ pigs, splicing from po*PLN*-WT exon 1 to the activated hu*PLN*-R14del exon 2 confirmed the induction of the R14del variant after Cre-mediated excision of the huPLN-WT/neo cassette (Fig. 11c).

## Discussion

Here we combined CRISPR-stimulated HR with recombinase-mediated cassette excision to establish a humanized knock-in pig model for PLN-mediated heart disease. While some of the GM approaches had been verified by us and others in earlier independent studies, their combination required careful design, advanced biotechnological methodologies, combinatorial breeding and thorough screening strategies to eventually achieve the desired full humanization of the *PLN* locus with a patient-relevant *PLN*^*huWT/huR14del*^ allelic constellation. The GM strategy for the PLN pig model was designed on several preconditions. On the one hand, while the arginine at position 13 of PLN is conserved between species, essential regulatory properties such as the fluctuation between mono- and pentameric structures^41^ and phosphorylation dynamics^42,43^ may differ between species owing to differences in amino acid sequences and post-transcriptional regulation (Fig. 1a,c). On the other hand, the high degree of conservation in SERCA proteins between mammals (Fig. 2) suggested that a completely humanized *PLN*^*huWT/huR14del*^ locus would recapitulate a patient-like Ca^2+^-regulation in cardiomyocytes. The proposed iGM strategy provided a fallback strategy in the event of impaired reproduction capabilities in mono-allelic *PLN*-R14del pigs (Fig. 3). In addition, it is also effective for developing two distinct genotypes from one targeting strategy. Consequently, laborious production and screening of SCCs are reduced, and numbers of costly and seasonally restricted SCNT experiments are minimized. The advanced iGM approach required robust HR and flexible RCME strategies.

Since the emergence of CRISPR–Cas9 technologies, GM in pigs has been effectively achieved by direct injection of CRISPR–Cas components into zygotes^35,44^, inducing DSBs to enable NHEJ-mediated insertions/deletions or homology-directed repair, using single-strand deoxy-nucleotides as repair templates for the defined mutations^34^. Site-directed placement of larger modifications, however, remains challenging and is commonly done in primary cells^36^, because cell clones can be verified for the desired modification before their use as nucleus donors in SCNT^38^. While most gene targeting attempts facilitate HR using vectors containing homologous arms of several 100 bp up to a few kilobases, we instead use BACs to create targeting vectors. The need for extensive screening of SCCs (Fig. 5) and GM pigs (Fig. 8) in BAC targeting is offset by the rapid and flexible bacterial recombineering in dedicated *Escherichia coli* strains^37^ and the use of extended regions of homology of several tens of kilobases. Either alone or in combination with CRISPR/Cas9, BAC vectors have facilitated HR at genomic sites that are considered silent in PKC such as *USH1C*^15^, *CFTR*^35^, *DMD*^34,35,45^ or *PLN*, hereby seemingly decoupling GM efficacy from chromatin accessibility^46,47^.

For the most part, RCME approaches in pigs have not relied on the numerous available Cre-expressing strains to crossbreed Cre-expressing and floxed cassette lines^21,48^. This is largely due to the inherent constraints of pig size and reproductive biology, which hinder the efficient exchange of GM strains and complex crossbreeding strategies commonly used in mice. Inspired by gene-therapy principles, vector-mediated delivery of Cre has emerged as practical alternative to induce RCME. This is particularly relevant for cancer research where mosaicism, resulting from incomplete distribution of gene-therapy vectors within a target tissue, becomes advantageous, reflecting the focal emergence and clonal expansion of tumor cells^49^. The oncopig^26–30^ is an outstanding example of an iGM model, in which the tumorigenic protein variants KRAS^G12D^ and TP53^R167H^ can be induced by localized Cre vector application in various tissues. This concept facilitated the investigation of cancer in the lung^42,43^, pancreas^50–54^, liver^55,56^ and brain^15^. In addition to specific administration protocols, the ability to control transgene induction by tailoring vector (sero-)types and promoters has enabled the optimization of oncogene activation in accordance with the physiological and anatomical conditions of the different target tissues. We made use of similar considerations here, increasing transduction of cardiomyocytes and Cre efficacy by coating the well-characterized AAV2/9 vectors with nanoparticles to substantially increase RCME frequencies in defined heart segments (Fig. 10). It is tempting to speculate that restricted local administration of vectors^57^ would further disseminate AAV-Cre vectors across the heart. Considering that many cardiomyopathies develop from focal areas, mosaic induction of hu*PLN*-R14del may still be sufficient to induce PLN-relevant disease signatures. This is particularly important for the PLN pig model if the evaluation of an accelerated phenotype in homozygous hu*PLN*^*huR14del/huR14del*^ animals is compromised by developmental deficits.

Nevertheless, mosaicism is undeniably associated with individual variations and concerns about the relevance of the pathogenic mechanisms found in such models for genetically inherited diseases. Injections of CRISPR–Cas components into mouse^58–60^, bovine^61^ or pig zygotes^62–64^ have shown that such considerations also generally apply to microinjection. However, the different mode of action and much faster kinetics of Cre recombinase reduce the risk of mosaicism^65^, which is in line with the observations from microinjection-derived piglets in our study (Fig. 9d,e). Thus, the microinjection protocol described here represents a pragmatic strategy for routine Cre-based iGM activation with low risk, although mosaicism cannot be completely ruled out in embryos derived from zygote injection. Moreover, embryos flushed from the oviducts of inseminated sows presumably exhibit higher developmental potential than embryos derived from SCNT or in vitro fertilization^61^.

Finally, applying Cre to cultivated primary cells represents another method to produce iGM pigs, using SCNT capabilities. Despite the reduced developmental potential of SCNT-derived embryos^66,67^, resulting in lower pregnancy rates, smaller litter sizes and impaired piglet vitality^68,69^, this approach allows the screening of defined cell populations before generating pigs of clonal identity (Fig. 5a). Beyond demanding extraordinary technical skills, SCNT efficiency critically depends on the yet undefined properties of the primary cells serving as the nuclear donors. The striking differences in proliferation and targeting capacities among distinct PKC lines (Fig. 5g) are in line with long-term experience and seem to correlate with SCNT success rates, as suggested by previous reports on the same PKCf^70^ and other PKC lines^57^. Tailored protocols and the use of larger cell populations at early passages during GM and clonal selection substantially improve the proliferative capacity of PKC, albeit not indefinitely^56^. This limits the screening capabilities on SCC (Fig. 5) and requires further evaluation in founder pigs and their offspring (Fig. 7). Optimization of lipofection nucleofection protocols, however, provides sufficient Cre-mediated iGM activation in small primary cell populations (Fig. 6), so that both hu*PLN*-WT and hu*PLN*-R14del variants were established already in the founder generation (Fig. 7c).

### Limitations of the study

We introduced a large GM into the pig genome by HR and subsequently applied Cre-mediated RCME with different methods. In line with animal welfare and cost considerations, we designed and executed a proof-of-concept study, limiting animal numbers and thereby preventing systematic comparison between Cre-applying methods. Although we explored different tissues with different methods, the frequency of mosaicism after Cre-MI into zygotes remains elusive and its occurrence cannot be eventually excluded. The work described here establishes a foundation for the systematic exploration of *PLN*-R14del cardiomyopathy; however, such studies need to be conducted comprehensively and carefully^71^, integrating complementary functional, structural and molecular approach at distinct stages of the disease.

## Conclusion

**Fig. 12:**
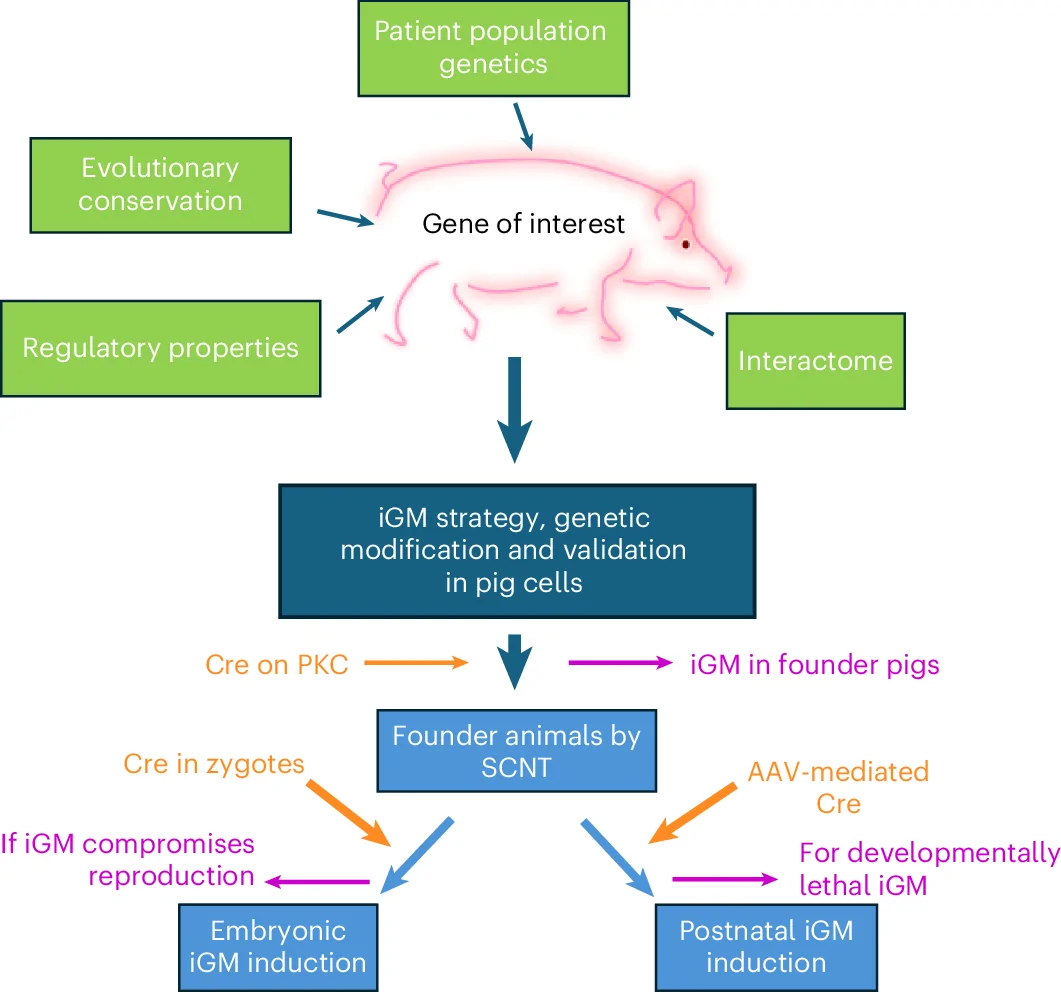
iGM in pigs. Planning of iGM strategy requires careful consideration of patient genetics, evolutionary conservation of the target gene, its regulation and its interactome. Depending on the intended application, iGM activation can be induced in SCCs before SCNT, in early embryos or postnatally.

Here we describe the comprehensive design of a pig model for the inherited arrhythmogenic *PLN*-R14del cardiomyopathy and outline effective strategies to achieve a fully humanized *PLN* locus using iGM approaches at distinct developmental stages. Each of the applied Cre-activation methods have their own merits and pitfalls that require careful consideration (Fig. 12). Our findings pave the way for novel future cGM approaches in pigs, but also in other species, particularly where the complementary transgenic Cre-driving lines are lacking or where their development, maintenance and distribution are challenging.

## Methods

### Regulatory statement

Work on blood or tissue samples of *PLN*-R14del patients took place at UMC Utrecht, under ethical regulations of the university. The collection and use of these human biological samples and accompanying clinical data were approved by the Biobank Research Ethics Committee (Toetsingscommissie Biobanken – TCBio) of UMC Utrecht under reference number 12–387 (UNRAVEL Biobank protocol). Written informed consent was explicitly obtained from all participating patients or their legal representatives before sample and data collection, allowing for their use in future medical research.

Animal work was conducted under the supervision of the responsible regulatory authorities: the Regierung von Oberbayern has approved animal experiments involving SCNT, maintenance of pigs and longitudinal monitoring at LMU Munich under the file number AZ 55.2-2532.Vet_02-17-136 and 55.2-2532.Vet_02-22-92. The State Veterinary Administration of the Czech Republic approved animal experiments on maintenance, sub-retinal intervention and longitudinal monitoring at IAPG Libechov under the experimental protocol numbers 75/2019 and 4190/2023.

### Animal background

Founder animals were generated on genetically modified primary cells of a female Swabian Hall pig. Conventional breeding was done by mating with hybrid pigs of agricultural background, dominated by German Landrace. This breeding program delivered experimental animals for AAV intervention in the F1 or downstream generations. Experiments on Cre-mRNA microinjection were done on zygotes delivered by matings of an F1 huPLN-WT boar with minipig sows of the Libechov breed.

### SNV validation in PLN carriers

The sequence of exon 2 of the human *PLN* gene (ENSG00000198523) was analyzed using whole-genome sequencing in patients who were carriers of the *PLN*-R14del variant (*n* = 46). gDNA was extracted from blood and used for Illumina library preparation, followed by paired-end sequencing (2 × 150 bp). Sequencing reads were aligned to the GRCh37/hg19 reference genome. On average, 928,344,367 reads were generated per sample, with a mean coverage of 35.85× ± 4.00 and a median coverage of 36.40× ± 4.08. The mean mappability was 99.76%. Variant calling was followed by annotation using dbSNP build 155 to identify known polymorphisms. Quality control procedures were applied throughout the pipeline to ensure adequate sequencing depth, high mappability and reliable variant detection.

### Bioinformatics evaluation

Protein sequences for mammalian PLN, SERCA1 and SERCA2 were extracted from GenBank (https://ncbi.nlm.nih.gov/). The genomic locus of mammalian PLN was identified using methods described previously^15^. In brief, BLAT search using human sequences of the *PLN* exons and the adjacent *CEP85L* exons were performed on the reference genome of macaque, marmoset, cattle, sheep, pig, horse, cat, dog, rabbit, mouse and rat (www.ensembl.org). The respective contig sequences were extracted aligned by a combination of DiAlign and CHAOS^75^. Proposed regulatory elements were extracted from the USCS Genome browser (https://genome.ucsc.edu/) as DNase sensitive elements^76^, ENCODE ChIP-Chip^77^, FANTOM5 enhancer elements^78^, GeneHancer elements^79^, FAIRE ENCODE regions^80^ and PreMod^81^. Any identified regulatory element was localized within the alignment in BioEdit (https://bioedit.software.informer.com/7.2/), and a density plot was created. Both a homology plot at nucleic acid level and a protein conservation plot were calculated in BioEdit based on the nucleotide summary assessment and entropy calculation functions, respectively. Coverage maps of the augmented alignment were plotted by JalView^82^.

### Targeting construct

BLAT search annotated the porcine *PLN* gene to pig chr1:43.5 Mb, embedded within the CEP85L gene, with identified exon corresponding GenBank no. NM_214213.1. Based on bioinformatics assessment, humanization was designed to encompass the entire PLN exon 2 (2,882 nt), along with 507 nt of the upstream intronic region and 501 bp of the downstream region. Two separate components were synthesized (BioCat gene synthesis service, Heidelberg, Germany) and assembled into the full modification vector by plasmid cloning. One element comprised a 5′ arm for bacterial recombineering (300 bp), restriction sites for *Nsi*I, *Nhe*I, *BamH*I and *Spe*I, a loxP site and a human sequence consisting of 2,879 nt of a defective *PLN* exon 2. This sequence included the causative *PLN* c.40_42delAGA mutation and the disease-allele specific SNPs (rs12198461 T > G, rs1051429 C > T), as well as 507-nt upstream intronic and 501-nt downstream sequences. *Not*I sites were placed at both sides of the constructs.

The second element contained 688 nt of intact human *PLN*-WT sequence localized between the naturally occurring and unique *Avr*II and *Pst*I sites. Using bacterial cloning, we first constituted a full hu*PLN*-WT cassette by replacing the defective PLN-R14del fragment between the *Avr*II/*Pst*I sites by the corresponding PLN-WT fragment. From this plasmid, an NsiI–hu*PLN*-WT–SpeI fragment was excised and coligated with an *Nhe*I–neo-loxP–*BamH*I fragment into an *Nsi*I-*BamH*I linearized hu*PLN*-R14del plasmid.

After verifying the finalized vector by restriction digest and sequencing, the entire modification element was excised by *NotI* and integrated into the porcine BAC CH242-318M5 (BACPAC Genomics), covering the pig *PLN* gene, by bacterial recombineering in bacterial strain SW106^83^. Correct modification of the BAC was confirmed according to established procedures^37^. This combined (1) endpoint PCR spanning the homologous arms, (2) BAC fingerprint analysis after *Nco*I digestion, confirming the correct localization of the modification, and (3) partial sequencing of critical regions on the BAC using primers huPLNup1r, huPLNf2, huPLNdo1f, neokanR and neokanF (Supplementary Information).

### Gene targeting and F0 founder pig generation

#### CRISPR–Cas gRNA

To stimulate homology-directed repair of the modified BAC vector within the target site in pig PKC lines^56^, CRISPR–Cas-mediated DSBs were introduced. To this end, the proposed target site in several PKC lines was amplified using the primer pair poPLN1f-poPLN1r (1,526 bp) and sequenced with primers poPLN5s and poPLN6s for naturally occurring SNPs. Three gRNAs (pln-BACg1, PLN-BACg2 and PLN-BACg3) were tested for their capacity to induce NHEJ mutations 48 h after nucleofection^36^, using the ICE CRISPR analysis tool of Sanger electropherograms (https://www.synthego.com/products/bioinformatics/analysis).

#### PKC GM

The high-potential cell line PKCf^12,15^ was co-nucleofected with the modified BAC (4,200 ng), plasmids expressing pln-BACg1 and pln-BACg3 under U6 promoter control (both 1,225 ng) and a plasmid encoding Cas9 (1,750 ng) in a 2b nucleofector (Amaxa by Lonza). SCCs were generated by seeding 100 cells per half area 96-well plates, selection with G41856 and propagation toward a 2× 96-well scale. One aliquot was used for analysis using the LoWA approach^36^, while the other served as a backup for potential SCNT. qPCR was carried out on a LightCycler96 (Roche Life Science) using FastStart Essential DNA Green Master (Roche Life Science) and the primer pair poPLN_q3f – poPLN_q3r, as well as the primer pair ush1c_qf1 – ush1c_qr1 for a reference site in the POU5F1 gene and ng_qf6 – ng_qr4 for a second reference in the NANOG gene. Then, candidate SCCs were examined by sequencing endpoint PCR amplicons, using poPLN1f-poPLN1r (1,526 bp) primers to detect the pig *PLN*-WT allele, huPLNdoF-neokanR (527 bp) and huPLN2F-huPLN1r (630 or 633 bp) primers to detect the human *PLN* WT, R14del exon 2 and the resistance cassette.

#### Cre treatment of SCC and SCNT

To activate the huR14del cassette in a subset of the cells, SCCs were treated with Cre recombinase using two approaches: renucleofection of a plasmid or lipofection of mRNA. For the latter, SCC aliquots were thawed, serum-starved for 48 h and then lipofected with 150 ng Cre mRNA (TransMessenger Lipofection kit, Qiagen) at a RNA:transfection reagent ratio of 1:7 for 1 h, after which the cells were directly used for SCNT.

To optimize nucleofection in small cell batches, aliquots of 5 × 10^4^ WT PKCs and unmodified SCC were nucleofected with 20 µl with 0.4 µg GFP-expressing plasmid (pMAX, Lonza) in 20 µl P2 Primary Cell Nucleofector Solution-Supplement 1 mixture, using different predefined programs in the 4D Nucleofector (Lonza). After pulsing, cells were suspended in 160 µl prewarmed culture medium and then seeded either immediately or after 10 min recovery at room temperature in 96-well plates. After reaching at least half-confluence, samples were trypsinized and then either directly fixed with 70% EtOH for 30 min at 4 °C for flow cytometry (FC) or fixed after spreading onto slides for confocal microscopy. For FC, cells were permeabilized (FIX&PERM Cell Permeabilization Kit, Invitrogen) and stained with DAPI. Measurements were performed using a Gallios FC (Beckman Coulter; 488 nm excitation and 530/30 nm bandpass emission filter for GFP laser) or a LSRFortessa Cell Analyzer (BD Biosciences; 405 nm excitation and 450/50 nm bandpass emission filter for DAPI, and 488 nm excitation and 530/30 nm bandpass emission filter for GFP).

Selected SCCs were then treated with 0.4 µg Cre-expressing plasmid, using either optimized conditions in the 4D nucleofector or standard conditions in a 2b nucleofector, then seeded in 96-well format and cryo-conserved as single aliquots for subsequent SCNT.

SCNT into enucleated oocytes and embryo transfer into synchronized gilts were performed according to standard procedures^38^. Birth of founder animals was induced in pregnant foster mothers by administration of Estrumate at day 115. (MSD Animal Health, Merck).

### Breeding, porcine zygote culture and microinjection

After reaching reproductive maturity, PLN F0 sows were inseminated with WT sperm. F1 offspring were genotyped and raised for breeding purposes or selected for AAV-Cre treatment (see below). After reaching fertility, one hu*PLN*-WT boar was used to inseminate synchronized gilts for ovary flushing. For this, estrus in Libechov minipigs was synchronized with 5 ml (4 mg/ml) Altrenogestum (Jensen) for 15 days. For superovulation, minipig gilts were stimulated with 500 IU of pregnant mare serum gonadotropin (Life-gene), 44 h after the last dose of Regumate. Eighty-four hours after pregnant mare serum gonadotropin injection, minipig gilts were stimulated with 500 IU of human chorionic gonadotropin. The next day, gilts were inseminated two times after estrus detection. Zygotes were collected by flushing the oviducts of inseminated gilts with prewarmed phosphate-buffered saline (PBS) at least twice. Collected zygotes were washed in M2 medium (M7167, Merck) and transferred to culture media. Embryos were cultured in a Defined Medium for Porcine Embryos (PZM-5, CSR-CK024, Cosmo Bio), supplemented with 1 mg/ml bovine serum albumin (Merck) at 39 °C with 5% CO_2_ and 5% O_2_. Zygotes were microinjected in M2 medium with approximately 10 pl of a 10 ng/μl StemMACS Cre Recombinase mRNA (130-101-113, Miltenyi Biotec) using a PM2000B microinjector (MicroData Instrument). The mRNA for microinjections was prepared according to the manufacturer’s protocol and stored at −80 °C. For in vitro assessment and embryo transfer, embryos were cultured in 50-μl culture media microdrops covered with mineral oil (Ovoil, Vitrolife).

### Laparoscopic transfer of the micromanipulated embryos (MME)

Laparoscopic transfer of MME was done under general anesthesia. To induce sedation, the minipigs were intramuscularly injected with a combination of agents: tiletamine 2 mg/kg + zolazepam 2 mg/kg (Zoletil 100, Virbac) + ketamine 2 mg/kg (Narketan 10, Chassot) + xylazine 0.4 mg/kg (Rometar 2%, Spofa). During anesthesia, heart rate and oxygen saturation (SpO2) were monitored. For analgesia during laparoscopic MME transfer, we injected the animals with Tramal (max. 400 mg/animal/day). Under deep sedation, the animals were intubated and put on artificial ventilation using an anesthesia machine (1 L/min oxygen + 1 L/min air) under isoflurane (ISOFLURIN 1,000 mg/g, Alvetra Werfft) anesthesia 1.5%. After shaving, cleaning and disinfecting the abdominal region of the animals with Braunol solution (B. Braun), a small incision (approximately 1–2 cm) was made at the umbilicus through the skin and subcutaneous tissue using a scalpel. Then, a Veress needle was inserted through the incision into the abdominal cavity and CO_2_ insufflation was performed to a pressure of 12 mm Hg. Next, the Veress needle was replaced with a laparoscopic trocar (Xion, 5 mm), and a rigid laparoscope with an angled optic (Xion, 5 mm) was introduced. Embryo transfer into the ampulla of oviducts of the recipient gilts was done using the laparoscopic tower (Xion). Approximately 10–20 cm from the first incision, in the caudolateral direction (left or right side, at a 45° angle from the midline), we performed two additional incisions for trocar placement and insertion of atraumatic graspers or other instruments. Completeness of ovulation and correct position of oviduct were checked using the laparoscopic screen station. After grasping the oviduct using an atraumatic grasper in the first and third part from ovarium, we introduced a handmade laparoscopic needle (18 G) through the opposite port and made a puncture in the oviduct next to the atraumatic grasper. Next, a catheter filled with MME (25–40 embryos) and cultivation medium was introduced through the laparoscopic needle into the oviduct lumen under visual guidance. After a short resting period (2–3 min) after MME deposition, the procedure was completed by removing the laparoscopic instruments and abdominal desufflation. Skin incisions were closed by nonresorbable material stitches and covered by Novikov solution.

### Tissue sampling for genotyping and primary cell isolation

Ear biopsies were taken from newborn animals, according to approved procedures under animal license regulation and stored at −20 °C. For isolation of pig PKC from founder animals and sampling of tissue from untreated pigs, animals were sedated with ketamine 100 mg/ml (Ursotamin, Serumwerk Bernburg) and azaperone 40 mg/ml (Stresnil, Elanco Animal Health), according to the manufacturer’s specifications. Fully anesthetized animals were euthanized by intravenous injection of T61 (MSD Animal Health). Comprehensive tissue sampling of the whole heart was performed immediately after euthanasia. Pig PKCs were isolated according to standard procedures^56^. Tissue for molecular analysis was frozen on dry ice and stored at −80 °C.

### Preparation and coating of AAV-Cre

Self-complementary rAAV pseudotype 2/9 particles encoding for Cre recombinase were produced via triple transfection as described previously^1^. In n brief, HEK 293T cells were cotransfected with the transgene construct encoding iCRE open reading frame (AAV2.CMV.iCRE.WPRE.SV40pA), a packaging plasmid with AAV2 rep and AAV9 cap genes and the pAdDeltaF6 helper plasmid (Addgene plasmid #112867, a gift from James M. Wilson) using PEI Max (Polysciences). Cells were collected after 72 h, and virus purification was carried out via iodixanol-gradient centrifugation, gravity-flow size-exclusion purification using Sephadex G100 SF resin (Sigma–Aldrich). Virus was concentrated in PBS using Amicon Ultra-15 centrifugal filter units (Merck), and titered by qPCR^84^ PMID 22428977). PAMAM-Peg-Cys modification and coating was carried out as described^85^: 18 µg of modified G2-PAMAM nanoparticles (Andrews ChemServices) in Opti-MEM I (Thermo Fisher Scientific) were added dropwise to 1 × 10^14^ vg of rAAVs and incubated for 30 min at room temperature, and stored for a maximum of 1 h on ice before application.

### Virus application and tissue sampling

Pigs with the huWT/poWT or huWT/huWT genotype were assigned to virus treatment or used as untreated controls. AAV-Cre administration was performed at 4–8 weeks of age (6.8–16.0 kg body weight) after sedating pigs via intramuscular injection of ketamine (15–20 mg/kg body weight) and azaperone (2 mg/kg body weight). To prevent inflammatory reactions and support hemodynamic stability, either dexamethasone (2 mg/kg body weight) or methylprednisolone (10 mg/kg body weight) was administered intravenously. The viral vector was administered systemically through a catheter into the caudal auricular vein (vena auricularis caudalis). Animals were then monitored for either 3 weeks (weight 15–23 kg) or 21–30 weeks (weight 73–94 kg). At the endpoints, pigs were premedicated (intramuscular ketamine 15–20 mg/kg, azaperone 2 mg/kg), followed by intratracheal intubation and intravenous anesthesia maintenance using propofol and continuous fentanyl infusion (2 µg/kg/h); anesthesia monitoring during echocardiography and left ventricular catheterization were performed in a subset of animals. In addition, electrophysiological studies were conducted in seven animals, including three virus-treated and four control pigs. Then, animals were euthanized by intracardiac injection of a saturated potassium chloride solution under deep anesthesia and heart, liver and blood samples were collected, immediately frozen on dry ice and stored at −80 °C.

In four animals, representative myocardial samples were collected from the left anterior descending artery region and from the circumflex artery region. In addition, liver samples and blood samples were collected for further analysis.

### Genotyping of piglets

gDNA was isolated from tail biopsies by using the Easy DNA kit (Invitrogen) DNAeasy kit (Qiagen), Macherey-Nagel Nucleospin (Macherey-Nagel) or Nexttec kits (Nexttec GmbH).

Genotyping of founder animals was performed via endpoint PCR using HotStart Taq polymerase (Qiagen) and a standard protocol of 5 min at 95 °C, 35× (20 s at 95 °C, 30 s at 8 °C, 1 min at 72 °C), 5 min at 72 °C, 5 min at 4 °C. Optional sequencing was done with amplification primers. Amplification with primers B2Mf-B2Mr (174 bp) was utilized to verify DNA quality. poPLN1f-poPLN1r (1,526 bp) detected the pig PLN-WT allele. huPLNdoF-neokanR (527 bp), neokanF-huPLNup1R (385 bp) and huPLNR14f-huPLN1r (421 bp) detected the modification between lox-sites and indicated the huPLN-WT genotype. huPLN2F-huPLN1r (630 versus 633 bp) and poPLN1f-huPLNup1r (812 bp) did not discriminate between the huPLN-WT and huPLN-R14del genotypes. All PCRs were run under standard conditions, except poPLN1f-poPLN1r, which required 2 min elongation time.

Genotyping of animals produced by breeding was done with primers GAPDHq1f-GAPDHq1r (351 bp) to verify DNA quality, poPLNq3f-poPLNq3r (112 bp) to determine the pig PLN allele, pln_qlf2-pln_qlr1 (116 bp) to determine the huPLN-R14del allele and neokanF-huPLNup1r (385 bp) to determine the human PLN-WT allele. All PCRs were performed with HotStart Taq polymerase, using a standard protocol of 5 min at 95 °C, 35× (20 s at 95 °C, 20 s at 58 °C, 30 s at 72 °C), 5 min at 72 °C, 5 min at 4 °C.

### Genotyping of embryos

Embryos were conserved after zygote injection and in vitro culture. Parthenogenetic porcine embryos were generated using in vitro matured oocytes^38,86^. In brief, 43–47 h after maturation, oocytes with expanded cumulus cells were denuded in hyaluronidase solution (1 mg/ml), and oocytes with an extruded first polar body were selected. The matured oocytes were washed twice in activation solution and then placed between two wire electrodes of a fusion chamber slide (CUY500G1, Nepa Gene) overlaid with activation solution. A single direct current pulse of 150 V/mm was applied for 100 µsec using an electrical pulsing machine (Multiporator, Eppendorf), followed by treatment with 5 µg/ml cytochalasin B for 3 h to suppress extrusion of the second polar body.

Embryos were cultured in Porcine Zygote medium-5^87^ in a humidified atmosphere of 5% CO_2_, 5% O_2_ and 90% N_2_ at 38.5 °C for 7 days. For isolation of embryonic gDNA, embryos were pretreated in a PBS solution containing 0.5 mg/ml of Pronase for 30–60 s until the zona pellucida was thickened. The reaction was stopped by transferring the embryos to PBS supplemented with 10% fetal calf serum. Embryos were then washed three times in serum-free PBS and individually placed in a lysis solution consisting of ddH_2_O containing 2 M MgCl_2_, 0.02 mg/ml Proteinase K, and 0.1% Triton X-100. Embryos were incubated in this solution for 1 h at 37 °C, followed by 8 min at 99 °C, and finally held at 4 °C for 15 min. Samples were vortexed for 10–15 s and briefly centrifuged. Two microliters of isolated DNA were used as a template in the first PCR. Taq 2xMaster Mix (NEB, #M0270L) was used following the manufacturer’s protocol, with primers at a final concentration of 1 µM.

Low copy numbers in embryos required two subsequent rounds of PCR (nested PCR). For establishing assays, gDNA of pigs carrying poPLN-WT, and huPLN-WT and huPLN-R14del variants were diluted to 10 copies/µl. In addition, DNA from parthenogenetic embryos was used to check for efficient DNA isolation from embryos. Two microliters of DNA were used as a template in the first PCR. Taq 2xMaster Mix (NEB, #M0270L) was used following the manufacturer’s protocol, with primers at a final concentration of 1 µM.

First PCR: 5 min at 95 °C, 15× (30 s at 95 °C, 30 s at 58 °C, 1 min at 72 °C), 5 min at 72 °C and cooled to 4 °C. Two microliters of the first nested PCR reaction was used as a template in the second nested PCR reaction.

Second PCR: 2 min at 95 °C, 35× (20 s at 95 °C, 20 s at 58 °C, 30 s at 72 °C), 5 min at 72 °C and cooled to 4 °C. For the PLN-R14del allele, the annealing was 64 °C. PCR products were visualized on agarose gels and bands were excised for verification by Sanger sequencing.

Porcine WT PLN was detected by the first primer pair poWT_ext-f – poWT_ext-r (690 bp) and second primer pair poWT_int-f – poWT_int-r (112 bp). Human full cassette was detected by the first primer pair huWT_ext-f – huWT_ext-r (544 bp) and second primer pair huWT_int-f – huWT_int-r (193 bp).

Human activated R14del was detected by the first primer pair huR14del_ext-f – huR14del_ext-r (380 bp) and second primer pair huR14del_int-f – huR14del_int-r (116 bp). Porcine B2M reference site was by the first primer pair poB2M_ext-f – poB2M_ext-r (451 bp) and second primer pair poB2M_int-f – poB2M_int-r (174 bp).

### GM copy number quantification

Copy numbers of po*PLN-*WT, hu-*PLN*-WT, activated hu-*PLN*-R14del and neo copies were determined by qPCR. For this, serial dilutions of a verified high quality gDNA were used as standard curve. Presuming that one haploid mammalian genome weighs 6.7 pg, DNA was diluted to 5,000 copies/µl and then further diluted into 2,500, 1,250, 625, 312.5, 156.25, 78.12 and 39.0 copies/µl. DNA isolated from PLN litters was diluted to approximately 2,500 copies/µl. Copy number ratios were determined in founder animals, F1 offspring and Cre-treated animals as described for LoWA, using ng_qf6-ng_qr4 (221 bp) and ush1c_qf1-ush1c_qr1 (90 bp) as genomic reference sites, poPLN_q3f-poPLN_q3r (112 bp) to detect pig *PLN* alleles, RESgDNA_q6f- RESgDNA_q6r (196 bp) to specifically determine the huPLN-WT cassette, pln_qlf2-pln_qlr1 (116 bp) to determine huPLN-R14del, and neo_prf1-neo_r32 (92 bp) to detect the copy numbers of the neo-selection cassette. Alternatively, Cre efficacy was determined by combining endpoint PCR with RE digestion. For this, a PCR product was amplified with LoxP_F2-loxP_R3 primers (897 bp in untreated copies, 894 bp in floxed cassettes) with Q5 HF polymerase (NEB, Ipswich), purified on agarose and treated with *Sap*I (NEB) to selectively cut amplicons from untreated GMs into 725 bp + 172 bp bands. Quantification was done by densitometry on pictures after separation on agarose gel, using ImageJ.

For detection of virus genomes after AAV-Cre treatment, qPCR was performed using primers CreF-CreR and the FastStart Universal SYBR Green Master Mix (Roche) with 10 min at 95 °C, 50× (15 s at 95 °C, 1 min at 60 °C), followed by a 65–95°C melting curve protocol (0.5 °C/min increment).

### Transcript analysis

Cryo-conserved tissue was powdered in two steps, first using a hammer and anvil then a mortar and pestle afterward. All instruments and samples were cooled in liquid nitrogen to avoid thawing of the samples during processing. Powdered tissue was transferred to a precooled tube and stored at −80 °C. RNA was isolated from powdered tissue samples using TRIzolTM (Invitrogen). Approximately 100 mg of powdered tissue was ground up in 1 ml of TRIzol by Polytron PT2500E (Kinematica) in a pulsatile manner to avoid overheating. Further steps were carried out as suggested by the TRIzol protocol and RNA was stored at −80 °C. For cDNA synthesis, samples were treated first with DNase I (Invitrogen) to remove possible contamination with gDNA. Then, RNA was reverse-transcribed into cDNA with SuperScriptTM III Reverse Transcriptase (Invitrogen).

All RT–PCRs were run under standard conditions 5 min at 95 °C, 35× (20 s at 94 °C, 20 s at 58 °C, 30 s at 72 °C), 5 min at 72 °C, 5 min at 4 °C, using HercII polymerase (Agilent). The primer pair GAPDHq1f-GAPDHq1r (181 bp) was used as positive control, pln_rt2f-pln_rt2r (594 bp) to detect poPLN transcripts and pln_rt3f-huPLN1r (567 bp) to detect humanized PLN transcripts. Sequencing was done with amplification primers.

qPCR assays were performed on LightCycler96 (Roche), using FastStart Essential DNA Green Master (Roche Life Science) and uracil-DNA glycosylase (Thermo Fisher Scientific). The standard cycler profile was 2 min at 50 °C, 10 min at 95 °C, 45× (10 s at 95 °C, 90 s at 63 °C), 10 s at 95 °C, 1 min at 60 °C, ramp 1 °C/s to 97 °C, 30 s at 37 °C. For quantification of PLN transcripts, primer pairs GAPDHq1f-GAPDHq1r (181 bp), ACTBq1f-ACTBq1r (96 bp), PPIAq1F-PPIAq1R (100 bp) and TBPq1f-TBPq1r (124 bp) as housekeeping genes, pln_qef1-pln_qer1 (112 bp) to detect humanized transcripts and pln_qef6-pln_qer6 (114 bp) to detect pig transcripts were used. All assays were run under standard conditions; sequencing was done with amplification primers.

In an alternative approach on PLN transcripts, the same GAPDHq1f-GAPDHq1r (181 bp) was used to normalize DELcDNA_q1f-DELcDNA_q1r (212 bp) to specifically amplify R14del transcripts, REScDNA_q1f-REScDNA_q1r (210 bp) to specifically amplify human WT transcripts and pln_qef8-pln_qer8 (181 bp) to specifically amplify PLN transcripts. All assays were run under standard conditions; sequencing was done with amplification primers.

### Reporting summary

Further information on research design is available in the Nature Portfolio Reporting Summary linked to this article.

## Online content

Any methods, additional references, Nature Portfolio reporting summaries, source data, extended data, supplementary information, acknowledgements, peer review information; details of author contributions and competing interests; and statements of data and code availability are available at https://doi.org/10.1038/s41684-026-01787-6.

## Supplementary information

Supplementary Information Oligo synthesis (pages 1–2), gene synthesis (pages 3–6) and Supplementary Fig. 1, including figure caption (pages 7–8).

Reporting Summary

## Source data

Source Data Fig. 4 BAC RE fingerprinting with enzyme; NcoI sample order indicated in pdf.

Source Data Fig. 7 Fig 7c: PCR-based genotyping of cloned and recloned founder piglets; Fig 7i: PCR-based genotyping of F1 piglets.

Source Data Fig. 9 Fig. 9a: genotyping of Cre-injected embryos derived from huPLN-WT × poWT matings; Fig 9b: genotyping of piglets derived from Cre-injected embryos.

Source Data Fig. 10 RE-induced fragment length polymorphism to determine postnatally activated R14del muations by Cre-AAV.

## Data Availability

The data that support the findings of this study are available from the corresponding authors upon request. Source data are provided with this paper.

## References

[1] Moretti, A. et al. Somatic gene editing ameliorates skeletal and cardiac muscle failure in pig and human models of Duchenne muscular dystrophy. *Nat. Med.* **26**, 207–214 (2020). 10.1038/s41591-019-0738-2 PMC7212064 31988462

[2] Wulkan, F. et al. Stem cell-derived cardiomyocytes expressing a dominant negative pacemaker HCN4 channel do not reduce the risk of graft-related arrhythmias. *Front. Cardiovasc. Med.* **11**, 1374881 (2024). 10.3389/fcvm.2024.1374881 PMC11263024 39045008

[3] Bähr, A. et al. Gene therapy for cardiomyopathy associated with Duchenne muscular dystrophy in a pig model. Prerprint at *bioRxiv* 10.1101/2023.10.02.560452 (2023).

[4] Boettcher, A. N., Schachtschneider, K. M., Schook, L. B. & Tuggle, C. K. Swine models for translational oncological research: an evolving landscape and regulatory considerations. *Mamm. Genome* **33**, 230–240 (2022). 10.1007/s00335-021-09907-y PMC8888764 34476572

[5] Holm, I. E., Alstrup, A. K. & Luo, Y. Genetically modified pig models for neurodegenerative disorders. *J. Pathol.* **238**, 267–287 (2016). 10.1002/path.4654 26446984

[6] Howland, D. et al. Large animal models of Huntington’s disease: what we have learned and where we need to go next. *J. Huntingtons Dis.* **9**, 201–216 (2020). 10.3233/JHD-200425 PMC7597371 32925082

[7] Watson, A. L., Carlson, D. F., Largaespada, D. A., Hackett, P. B. & Fahrenkrug, S. C. Engineered swine models of cancer. *Front. Genet.* **7**, 78 (2016). 10.3389/fgene.2016.00078 PMC4860525 27242889

[8] Klymiuk, N. et al. Tailored pig models for preclinical efficacy and safety testing of targeted therapies. *Toxicol. Pathol.* **44**, 346–357 (2016). 10.1177/0192623315609688 26511847

[9] Tanihara, F., Hirata, M. & Otoi, T. Current status of the application of gene editing in pigs. *J. Reprod. Dev.* **67**, 177–187 (2021). 10.1262/jrd.2021-025 PMC8238678 33840678

[10] Wu, H. et al. Engineering CRISPR/Cpf1 with tRNA promotes genome editing capability in mammalian systems. *Cell. Mol. Life Sci.* **75**, 3593–3607 (2018). 10.1007/s00018-018-2810-3 PMC11105780 29637228

[11] Jin, Y. X. et al. Production of pigs expressing a transgene under the control of a tetracycline-inducible system. *PLoS ONE* **9**, e86146 (2014). 10.1371/journal.pone.0086146 PMC3893280 24454957

[12] Klymiuk, N. et al. First inducible transgene expression in porcine large animal models. *FASEB J.* **26**, 1086–1099 (2012). 10.1096/fj.11-185041 22138035

[13] Anand, R. P. et al. Design and testing of a humanized porcine donor for xenotransplantation. *Nature* **622**, 393–401 (2023). 10.1038/s41586-023-06594-4 PMC10567564 37821590

[14] Griffith, B. P. et al. Transplantation of a genetically modified porcine heart into a live human. *Nat. Med.* **31**, 589–598 (2025). 10.1038/s41591-024-03429-1 39779924

[15] Grotz, S. et al. Early disruption of photoreceptor cell architecture and loss of vision in a humanized pig model of usher syndromes. *EMBO Mol. Med.* **14**, e14817 (2022). 10.15252/emmm.202114817 PMC8988205 35254721

[16] Oumard, A., Qiao, J., Jostock, T., Li, J. & Bode, J. Recommended method for chromosome exploitation: RMCE-based cassette-exchange systems in animal cell biotechnology. *Cytotechnology* **50**, 93–108 (2006). 10.1007/s10616-006-6550-0 PMC3476001 19003073

[17] Turan, S., Zehe, C., Kuehle, J., Qiao, J. & Bode, J. Recombinase-mediated cassette exchange (RMCE)—a rapidly-expanding toolbox for targeted genomic modifications. *Gene* **515**, 1–27 (2013). 10.1016/j.gene.2012.11.016 23201421

[18] Kuhn, R., Schwenk, F., Aguet, M. & Rajewsky, K. Inducible gene targeting in mice. *Science* **269**, 1427–1429 (1995). 10.1126/science.7660125 7660125

[19] Schebelle, L. et al. Efficient conditional and promoter-specific in vivo expression of cDNAs of choice by taking advantage of recombinase-mediated cassette exchange using FlEx gene traps. *Nucleic Acids Res.* **38**, e106 (2010). 10.1093/nar/gkq044 PMC2875000 20139417

[20] Kameyama, Y., Kawabe, Y., Ito, A. & Kamihira, M. An accumulative site-specific gene integration system using Cre recombinase-mediated cassette exchange. *Biotechnol. Bioeng.* **105**, 1106–1114 (2010). 10.1002/bit.22619 19998287

[21] Li, S. et al. Dual fluorescent reporter pig for Cre recombination: transgene placement at the ROSA26 locus. *PLoS ONE* **9**, e102455 (2014). 10.1371/journal.pone.0102455 PMC4099177 25025770

[22] Li, X. et al. Rosa26-targeted swine models for stable gene over-expression and Cre-mediated lineage tracing. *Cell Res.* **24**, 501–504 (2014). 10.1038/cr.2014.15 PMC3975497 24503648

[23] Wang, K. et al. Cre-dependent Cas9-expressing pigs enable efficient in vivo genome editing. *Genome Res.* **27**, 2061–2071 (2017). 10.1101/gr.222521.117 PMC5741047 29146772

[24] Li, S. et al. Viable pigs with a conditionally-activated oncogenic KRAS mutation. *Transgenic Res.* **24**, 509–517 (2015). 10.1007/s11248-015-9866-8 25716163

[25] Schook, L. B. et al. A genetic porcine model of cancer. *PLoS ONE* **10**, e0128864 (2015). 10.1371/journal.pone.0128864 PMC4488487 26132737

[26] Kalla, D. et al. The missing link: Cre pigs for cancer research. *Front. Oncol.* **11**, 755746 (2021). 10.3389/fonc.2021.755746 PMC8531543 34692545

[27] Luo, W. et al. Generation of AQP2-Cre transgenic mini-pigs specifically expressing Cre recombinase in kidney collecting duct cells. *Transgenic Res.* **23**, 365–375 (2014). 10.1007/s11248-013-9774-8 24307331

[28] Song, Y. et al. Germ cell-specific expression of Cre recombinase using the VASA promoter in the pig. *FEBS Open Bio.* **6**, 50–55 (2016). 10.1002/2211-5463.12005 PMC4794798 27047735

[29] Chen, L. et al. Construction of transgenic swine with induced expression of Cre recombinase. *Animal* **4**, 767–771 (2010). 10.1017/S1751731109991571 22444131

[30] Oh, D. et al. Generation of a genetically engineered porcine melanoma model featuring oncogenic control through conditional Cre recombination. *Sci. Rep.* **15**, 1616 (2025). 10.1038/s41598-024-82554-w PMC11724099 39794352

[31] Nagel, B. et al. Left ventricular hypertrabeculation/noncompaction with epilepsy, other heart defects, minor facial anomalies and new copy number variants. *BMC Med. Genet.* **13**, 60 (2012). 10.1186/1471-2350-13-60 PMC3490829 22830313

[32] van der Zwaag, P. A. et al. Recurrent and founder mutations in the Netherlands-Phospholamban p.Arg14del mutation causes arrhythmogenic cardiomyopathy. *Neth. Heart J.* **21**, 286–293 (2013). 10.1007/s12471-013-0401-3 PMC3661879 23568436

[33] Eijgenraam, T. R. et al. The phospholamban p.(Arg14del) pathogenic variant leads to cardiomyopathy with heart failure and is unreponsive to standard heart failure therapy. *Sci. Rep.* **10**, 9819 (2020). 10.1038/s41598-020-66656-9 PMC7300032 32555305

[34] Klymiuk, N. et al. Dystrophin-deficient pigs provide new insights into the hierarchy of physiological derangements of dystrophic muscle. *Hum. Mol. Genet.* **22**, 4368–4382 (2013). 10.1093/hmg/ddt287 23784375

[35] Klymiuk, N. et al. Sequential targeting of CFTR by BAC vectors generates a novel pig model of cystic fibrosis. *J. Mol. Med.* **90**, 597–608 (2012). 10.1007/s00109-011-0839-y 22170306

[36] Vochozkova, P., Simmet, K., Jemiller, E. M., Wunsch, A. & Klymiuk, N. Gene editing in primary cells of cattle and pig. *Methods Mol. Biol.* **1961**, 271–289 (2019). 10.1007/978-1-4939-9170-9_17 30912052

[37] Auch, H., Klymiuk, N. & Runa-Vochozkova, P. Modifying bacterial artificial chromosomes for extended genome modification. *Methods Mol. Biol.* **2495**, 67–90 (2022). 10.1007/978-1-0716-2301-5_4 35696028

[38] Kurome, M., Kessler, B., Wuensch, A., Nagashima, H. & Wolf, E. Nuclear transfer and transgenesis in the pig. *Methods Mol. Biol.* **1222**, 37–59 (2015). 10.1007/978-1-4939-1594-1_4 25287337

[39] Brinster, R. L., Chen, H. Y., Trumbauer, M. E., Yagle, M. K. & Palmiter, R. D. Factors affecting the efficiency of introducing foreign DNA into mice by microinjecting eggs. *Proc. Natl Acad. Sci. USA* **82**, 4438–4442 (1985). 10.1073/pnas.82.13.4438 PMC391116 3892534

[40] Whitworth, K. M. et al. Zygote injection of RNA encoding Cre recombinase results in efficient removal of LoxP flanked neomycin cassettes in pigs. *Transgenic Res.* **27**, 167–178 (2018). 10.1007/s11248-018-0064-3 29516259

[41] Hamze, J. G., Cambra, J. M., Navarro-Serna, S. & Martinez-Serrano, C. A. Navigating gene editing in porcine embryos: Methods, challenges, and future perspectives. *Genomics* **117**, 111014 (2025). 10.1016/j.ygeno.2025.111014 39952413

[42] Vafiadaki, E., Haghighi, K., Arvanitis, D. A., Kranias, E. G. & Sanoudou, D. Aberrant PLN–R14del protein interactions intensify SERCA2a inhibition, driving impaired Ca^2+^ handling and arrhythmogenesis. *Int. J. Mol. Sci.* 10.3390/ijms23136947 (2022). PMC9266971 35805951

[43] Vostrikov, V. V., Soller, K. J., Ha, K. N., Gopinath, T. & Veglia, G. Effects of naturally occurring arginine 14 deletion on phospholamban conformational dynamics and membrane interactions. *Biochim. Biophys. Acta* **1848**, 315–322 (2015). 10.1016/j.bbamem.2014.09.007 PMC4258429 25251363

[44] Rogers, C. S. et al. Disruption of the CFTR gene produces a model of cystic fibrosis in newborn pigs. *Science* **321**, 1837–1841 (2008). 10.1126/science.1163600 PMC2570747 18818360

[45] Beshr, G. et al. Photorhabdus luminescens lectin A (PllA): a new probe for detecting alpha-galactoside-terminating glycoconjugates. *J. Biol. Chem.* **292**, 19935–19951 (2017). 10.1074/jbc.M117.812792 PMC5712630 28972138

[46] Chari, R., Mali, P., Moosburner, M. & Church, G. M. Unraveling CRISPR–Cas9 genome engineering parameters via a library-on-library approach. *Nat. Methods* **12**, 823–826 (2015). 10.1038/nmeth.3473 PMC5292764 26167643

[47] Jensen, K. T. et al. Chromatin accessibility and guide sequence secondary structure affect CRISPR–Cas9 gene editing efficiency. *FEBS Lett.* **591**, 1892–1901 (2017). 10.1002/1873-3468.12707 28580607

[48] Moon, J. et al. Production of porcine cloned embryos derived from cells conditionally expressing an exogenous gene using Cre-loxP. *Zygote* **20**, 423–425 (2012). 10.1017/S0967199411000773 22272567

[49] Schook, L. B. et al. Emerging technologies to create inducible and genetically defined porcine cancer models. *Front. Genet.* **7**, 28 (2016). 10.3389/fgene.2016.00028 PMC4770043 26973698

[50] Funk, F. et al. Phospholamban pentamerization increases sensitivity and dynamic range of cardiac relaxation. *Cardiovasc. Res.* **119**, 1568–1582 (2023). 10.1093/cvr/cvad037 PMC10318394 36869774

[51] Glaves, J. P., Primeau, J. O., Espinoza-Fonseca, L. M., Lemieux, M. J. & Young, H. S. The phospholamban pentamer alters function of the sarcoplasmic reticulum calcium pump SERCA. *Biophys. J.* **116**, 633–647 (2019). 10.1016/j.bpj.2019.01.013 PMC6383005 30712785

[52] Smeazzetto, S. et al. Conformational memory in the association of the transmembrane protein phospholamban with the sarcoplasmic reticulum calcium pump SERCA. *J. Biol. Chem.* **292**, 21330–21339 (2017). 10.1074/jbc.M117.794453 PMC5766962 29081402

[53] Traaseth, N. J. et al. Spectroscopic validation of the pentameric structure of phospholamban. *Proc. Natl Acad. Sci. USA* **104**, 14676–14681 (2007). 10.1073/pnas.0701016104 PMC1976191 17804809

[54] Wittmann, T., Lohse, M. J. & Schmitt, J. P. Phospholamban pentamers attenuate PKA-dependent phosphorylation of monomers. *J. Mol. Cell. Cardiol.* **80**, 90–97 (2015). 10.1016/j.yjmcc.2014.12.020 25562800

[55] Kurome, M. et al. Factors influencing the efficiency of generating genetically engineered pigs by nuclear transfer: multi-factorial analysis of a large data set. *BMC Biotechnol.* **13**, 43 (2013). 10.1186/1472-6750-13-43 PMC3691671 23688045

[56] Richter, A. et al. Potential of primary kidney cells for somatic cell nuclear transfer mediated transgenesis in pig. *BMC Biotechnol.* **12**, 84 (2012). 10.1186/1472-6750-12-84 PMC3537537 23140586

[57] Stirm, M. et al. Systemic deletion of DMD exon 51 rescues clinically severe Duchenne muscular dystrophy in a pig model lacking DMD exon 52. *Proc. Natl Acad. Sci. USA* **120**, e2301250120 (2023). 10.1073/pnas.2301250120 PMC10629550 37428903

[58] Isom, S. C., Li, R. F., Whitworth, K. M. & Prather, R. S. Timing of first embryonic cleavage is a positive indicator of the in vitro developmental potential of porcine embryos derived from in vitro fertilization, somatic cell nuclear transfer and parthenogenesis. *Mol. Reprod. Dev.* **79**, 197–207 (2012). 10.1002/mrd.22013 22213403

[59] Yamanaka, K., Sugimura, S., Wakai, T., Kawahara, M. & Sato, E. Difference in sensitivity to culture condition between in vitro fertilized and somatic cell nuclear transfer embryos in pigs. *J. Reprod. Dev.* **55**, 299–304 (2009). 10.1262/jrd.20174 19293559

[60] Zhai, Y. et al. Profiling the transcriptomic signatures and identifying the patterns of zygotic genome activation - a comparative analysis between early porcine embryos and their counterparts in other three mammalian species. *BMC Genomics* **23**, 772 (2022). 10.1186/s12864-022-09015-4 PMC9700911 36434523

[61] Wei, J. et al. Cytoplasmic injection of zygotes to genome edit naturally occurring sequence variants into bovine embryos. *Front. Genet.* **13**, 925913 (2022). 10.3389/fgene.2022.925913 PMC9310181 35899192

[62] Bauer, B. K. et al. Transcriptional profiling by deep sequencing identifies differences in mRNA transcript abundance in in vivo-derived versus in vitro-cultured porcine blastocyst stage embryos. *Biol. Reprod.* **83**, 791–798 (2010). 10.1095/biolreprod.110.085936 20668257

[63] Cambra, J. M., Martinez, E. A., Rodriguez-Martinez, H., Gil, M. A. & Cuello, C. Transcriptional profiling of porcine blastocysts produced in vitro in a chemically defined culture medium. *Animals*10.3390/ani11051414 (2021). PMC8156047 34069238

[64] Chen, P. R. et al. Production of pigs from porcine embryos generated in vitro. *Front. Anim. Sci.* 10.3389/fanim.2022.826324 (2022).

[65] Hashimoto, M., Yamashita, Y. & Takemoto, T. Electroporation of Cas9 protein/sgRNA into early pronuclear zygotes generates non-mosaic mutants in the mouse. *Dev. Biol.* **418**, 1–9 (2016). 10.1016/j.ydbio.2016.07.017 27474397

[66] Navarro-Serna, S. et al. Generation of nonmosaic, two-pore channel 2 biallelic knockout pigs in one generation by CRISPR–Cas9 microinjection before oocyte insemination. *CRISPR J.* **4**, 132–146 (2021). 10.1089/crispr.2020.0078 33616447

[67] Whitworth, K. M. et al. Zygote injection of CRISPR/Cas9 RNA successfully modifies the target gene without delaying blastocyst development or altering the sex ratio in pigs. *Transgenic Res.* **26**, 97–107 (2017). 10.1007/s11248-016-9989-6 PMC5247313 27744533

[68] Araki, K., Araki, M., Miyazaki, J. & Vassalli, P. Site-specific recombination of a transgene in fertilized eggs by transient expression of Cre recombinase. *Proc. Natl Acad. Sci. USA* **92**, 160–164 (1995). 10.1073/pnas.92.1.160 PMC42837 7816809

[69] Luckow, B. et al. Microinjection of Cre recombinase protein into zygotes enables specific deletion of two eukaryotic selection cassettes and enhances the expression of a DsRed2 reporter gene in Ccr2/Ccr5 double-deficient mice. *Genesis* **47**, 545–558 (2009). 10.1002/dvg.20531 19517561

[70] Lampela, J. et al. Caridac vein retroinjections provide an efficient approach for global left ventricular gene transfer with adenovirus and adeno-associated virus. *Sci. Rep.* **14**, 1467 (2024). 10.1038/s41598-024-51712-5 PMC10794695 38233585

[71] Jaudas, F. et al. Perinatal dysfunction of innate immunity in cystic fibrosis. *Sci. Transl. Med.* **17**, eadk9145 (2025). 10.1126/scitranslmed.adk9145 39841805

[72] Seidel, K. et al. Structural characterization of Ca(2+)-ATPase-bound phospholamban in lipid bilayers by solid-state nuclear magnetic resonance (NMR) spectroscopy. *Biochemistry* **47**, 4369–4376 (2008). 10.1021/bi7024194 18355039

[73] Gorski, P. A., Trieber, C. A., Ashrafi, G. & Young, H. S. Regulation of the sarcoplasmic reticulum calcium pump by divergent phospholamban isoforms in zebrafish. *J. Biol. Chem.* **290**, 6777–6788 (2015). 10.1074/jbc.M114.585604 PMC4358104 25593315

[74] Primeau, J. O., Armanious, G. P., Fisher, M. E. & Young, H. S. The sarcoendoplasmic reticulum calcium ATPase. *Subcell. Biochem.* **87**, 229–258 (2018). 10.1007/978-981-10-7757-9_8 29464562

[75] Brudno, M., Steinkamp, R. & Morgenstern, B. The CHAOS/DIALIGN WWW server for multiple alignment of genomic sequences. *Nucleic Acids Res.* **32**, W41–W44 (2004). 10.1093/nar/gkh361 PMC441499 15215346

[76] Thurman, R. E. et al. The accessible chromatin landscape of the human genome. *Nature* **489**, 75–82 (2012). 10.1038/nature11232 PMC3721348 22955617

[77] Wang, J. et al. Factorbook.org: a Wiki-based database for transcription factor-binding data generated by the ENCODE consortium. *Nucleic Acids Res.* **41**, D171–D176 (2013). 10.1093/nar/gks1221 PMC3531197 23203885

[78] Andersson, R. et al. An atlas of active enhancers across human cell types and tissues. *Nature* **507**, 455–461 (2014). 10.1038/nature12787 PMC5215096 24670763

[79] Fishilevich, S. et al. GeneHancer: genome-wide integration of enhancers and target genes in GeneCards. *Database*10.1093/database/bax028 (2017). PMC5467550 28605766

[80] Giresi, P. G., Kim, J., McDaniell, R. M., Iyer, V. R. & Lieb, J. D. FAIRE (Formaldehyde-Assisted Isolation of Regulatory Elements) isolates active regulatory elements from human chromatin. *Genome Res.* **17**, 877–885 (2007). 10.1101/gr.5533506 PMC1891346 17179217

[81] Ferretti, V. et al. PReMod: a database of genome-wide mammalian *cis*-regulatory module predictions. *Nucleic Acids Res.* **35**, D122–D126 (2007). 10.1093/nar/gkl879 PMC1761432 17148480

[82] Waterhouse, A. M., Procter, J. B., Martin, D. M., Clamp, M. & Barton, G. J. Jalview Version 2—a multiple sequence alignment editor and analysis workbench. *Bioinformatics* **25**, 1189–1191 (2009). 10.1093/bioinformatics/btp033 PMC2672624 19151095

[83] Warming, S., Costantino, N., Court, D. L., Jenkins, N. A. & Copeland, N. G. Simple and highly efficient BAC recombineering using galK selection. *Nucleic Acids Res.* **33**, e36 10.1093/nar/gni035 (2005). PMC549575 15731329

[84] Aurnhammer, C. et al. Universal real-time PCR for the detection and quantification of adeno-associated virus serotype 2-derived inverted terminal repeat sequences. *Hum. Gene Ther. Methods* **23**, 18–28 10.1089/hgtb.2011.034 (2012). 22428977

[85] Bozoglu, T. et al. Endothelial retargeting of AAV9 in vivo. *Adv. Sci.* **9**, e2103867 (2022). 10.1002/advs.202103867 PMC8895123 35023328

[86] Yuan, Y. et al. Quadrupling efficiency in production of genetically modified pigs through improved oocyte maturation. *Proc. Natl Acad. Sci. USA* **114**, E5796–E5804 (2017). 10.1073/pnas.1703998114 PMC5530680 28673989

[87] Yoshioka, K., Suzuki, C. & Onishi, A. Defined system for in vitro production of porcine embryos using a single basic medium. *J. Reprod. Dev.* **54**, 208–213 (2008). 10.1262/jrd.20001 18408352

