# Supplementary material for: Activating a patient-relevant pathogenic variant of a genetically defined heart disease in a humanized pig model
Source: Lab Anim (NY). 2026 Sep 11;55(10):386–401. doi: 10.1038/s41684-026-01787-6 (PMC13619452; doi:10.1038/s41684-026-01787-6)
Supplement: Supplementary file 1 [file 41684_2026_1787_MOESM1_ESM.pdf]

lab animal

https://doi.org/10.1038/s41684-026-01787-6

Activating a patient-relevant pathogenic variant of a genetically defined heart disease in a humanized pig model

In the format provided by the authors and unedited

Oligo & Gene Synthesis:
Primers (with RE recognition sites underlined):
Construct verification:

- poPLN1f:	5´- AAGGAATGGCAAACATTTCA
- poPLN1r:	5´- GGAGTCAGTGGGCTATTTCA
-poPLN5s:	5´- CATCTTATTCCAGAAATGC
-poPLN6s:	5´- TGAGGTATTGGACTTTATC
-poPLNup1f:	5´- TGCAAATGGAAACGCTGTTC
-poPLNdo1r:	5´- CAAACTCACAGTCCCTCCAC
-huPLNup1r:	5´- CACACCATGACCGTCTCATT
-huPLNf2:	5´- TGCTGAGGAAGATGAATTAGTG
-huPLNdo1f:	5´- CCCAAGTCCAGACACACAAG
-neokanR:	5´- GTGGATGTGGAATGTGTGC
-neokanF:	5´- CACACCATGACCGTCTCATT

LoWA - qPCR:

- poPLN_q3f:	5´-GGTTTCACACTGATAGAGGGATT
- poPLN_q3r:	5´-ACCAGTAAAGCAGTTGTCTCAT
- ng_qf6:	5´-GAGGCTTCACTTGTTAAGGG
- ng_qr4:	5´-CTGAGATCAGGGTAGACATAC
- ush1c_q1f:	5'- CTTCTTGGAGCAGATGGGATAAA
- ush1c_q1r:	5'- GAATGCAGATTTCTGGTTTCCAC

SCNT founder and breeding genotyping and copy number determination:

- poPLN1f:	5´- AAGGAATGGCAAACATTTCA
- poPLN1r:	5´- GGAGTCAGTGGGCTATTTCA
- huPLNdo1f:	5´- CCCAAGTCCAGACACACAAG
- neokanR:	5´- GTGGATGTGGAATGTGTGC
- huPLN2F:	5´- TGCTGAGGAAGATGAATTAGTG
- huPLN1r:	5´- TGGGATAGAAATTTGTGAGCCA
- neokanF:	5´- CACACCATGACCGTCTCATT
- huPLNR14f:	5´- CACTCGCTCAGCTATAAGAAGA
- neoPf:	5´- CAGCTGTGCTCGACGTTGTC
- neoSr:	5´- GAGTCAACTAGTCCTCAGAAGAACTCGTCAAG
- GAPDHq1f:	5´- CAGAACATCATCCCTGCTTC
- GAPDHq1r:	5´- GCTTCACCACCTTCTTGATG
- huPLNup1rf:	5´- CACACCATGACCGTCTCATT
- pln_qlf2:	5´- GAACAGCCCAAAACAAGCTGC
- pln_qlr1:	5´- CAATAAAGACCCCAAGGATCCAT
- poPLN_q3f:	5´- GGTTTCACACTGATAGAGGGATT
- poPLN_q3r:	5´- ACCAGTAAAGCAGTTGTCTCAT
- RESgDNA_q6f:	5´- GGGAGGATTGGGAAGACAAT
- RESgDNA_q6r:	5´- CTGGTCACAAAAACATCACCA
- neo_prf1:	5´- CCATTGAACAAGATGGATTGCAC
- neo_r32:	5´- CATCAGAGCAGCCGATTGTCTGTTG
- CreF:	5´- GGTGCAAGCTGAACAACAGG
- CreR:	5´- AGCATTGGAGTCAGAAGGGC
- LoxP_F2:	5´- CCAAAACAAGCTGCCCCCTAAA
- LoxP_R3:	5´- TTAAGCTGATGTGGCAAGCTGC

Zygote nested PCR genotyping:

- poWT_ext-f: 5´- TCAACATAAGTGCTGCTAACAAAG
- poWT_ext-r: 5´- ACGTAGAAGGCGACATTAAACA
- poWT_int-f: 5´- GGTTTCACACTGATAGAGGGATT
- poWT_int-r: 5´- ACCAGTAAAGCAGTTGTCTCAT
- huWT_ext-f: 5´- CTCCCACTGTCCTTTCCTAATAA
- huWT_ext-r: 5´- GGAGTAATCCTCAGTGTCTTCC
- huWT_int-f: 5´- GGGAGGATTGGGAAGACAAT
- huWT_int-r: 5´- CTGGTCACAAAAACATCACCA
- huR14del_ext-f: 5´- CATCTTATTCCAGAAATGCTCAGAA
- huR14del_ext-r: 5´- ACCCTAGGCTGATTGATGTAAA
- huR14del_int-f: 5´- GAACAGCCCAAAACAAGCTGC
- huR14del_int-r: 5´- CAATAAAGACCCCAAGGATCCAT
- poB2M_ext-f: 5´- ATCCTCTGGCGTTTCATCTG
- poB2M_ext-r: 5´- GTGCTACTAGACGGAGCTTTAAT
- poB2M_int-f: 5´- CGGAAAGCCAAATTACCTGA
- poB2M_int-r: 5´- TCCACAGCGTTAGGAGTGAA

Transcripts (RT-PCR, RT-qPCR):

- GAPDHq1f: 5´- CAGAACATCATCCCTGCTTC
- GAPDHq1r: 5´- GCTTCACCACCTTCTTGATG
- TBPq1f: 5´- GATGGACGTTCGGTTTAGG
- TBPq1r: 5´- AGCAGCACAGTACGAGCAA
- PPIAq1f: 5´- CTGAAGCATACGGGTCCTGG
- PPIAq1r: 5´- CCAACCACTCAGTCTTGGCA
- ACTBq1f: 5´- AGCGCAAGTACTCCGTGTG
- ACTBq1r: 5´- CGGACTCATCGTACTCCTGCTT
- pln_rt2f: 5´- ACACCGATAAGACTTCATACAACT
- pln_rt2r: 5´- TGTAGGTTCTGATACCACTAGAGA
- pln_rt3f: 5’- CTGTGATCATCATAAACAGCCAAG
- huPLN1r: 5’- TGGGATAGAAATTTGTGAGCCA
- pln_qef1: 5’- GAAGAACAGAAGGGCAGAGATT
- pln_qer1: 5’- AAACCAGTGAGGTGAGTGTATC
- pln_qef6: 5’- GGAACAGAGGGAAAAGGGGTT
- pln_qer6: 5’- ACTGCCACCAGTAAAGCAGTT
- pln_qef8: 5’- ACGGTTTGCTAAAGTATGGTTAC
- pln_qer8: 5’- CTTTGCTCAAGAAATCCCTCTATC
- DELcDNA_q1f: 5´- CTTTCCAGCTAAACACCGATA
- DELcDNA_q1r: 5´- TTTCAATGGTTGAGGCTCTTAT
- REScDNA_q1f: 5´- TCCAGCTAAACACCGATAAGAC
- REScDNA_q1r: 5´- TCAATGGTTGAGGCTCTTCTT

gRNAs (PAM underlined):

- PLNg1: 5´-gtatctcaatagtctaTGtgTGG
- PLNg2: 5´-cacatagactattgagATacTGG
- PLNg3: 5´-catctagtcattctgaGAgaAGG

Modification vector:

5´- homologous arm	1-300
loxP-site	328-361
PLN rescue cassette	362-4260
Including human PLN WT exon 2	*869-3759*
Including human PLN WT coding sequence	*966-1124*
Neomycin resistance cassette	4266-5992
loxP-site	5993-6026
PLN rescue cassette	6033-9921
Including human PLN WT exon 2	*6533-9420*
Including human PLN WT coding sequence	*6630-6785*
3´-homologous arm	9928-10227

*Nsi*I (atgcat), *Nhe*I (gctagc), *BamH*I (ggatcc), *Avr*II (cctagg), *Pst*I (ctgcag), and *Spe*I (actagt) restriction enzyme sites are underlined.

```
   1 gcttcagaaa atctatacta aaatctcaaa acatgtagat gtgaataaat gtaaactctt
  61 caaatgcgct ttatttttac atcttattcc agaaatgctc agaaataaaa agaatatctc
 121 tttgaaacag tatctaatgt catacttaaa tgcactcatt tctttttatt agattgctct
 181 ggagccatta atacactaat gttcacaaca attatgctca tagagaaacg gtcttaatga
 241 aactgtatgt aagaacttgc ctaagggaac agcccaaaac aagctgcccc ctaaactaaa
 301 atgcatgaca ataaacgcta gctggcaATA ACTTCGTATA ATGTATGCTA TACGAAGTTA
 361 Tggatcctt ggggtcttta ttgagaagtt tggtgatgtt tttgtgacca gaaatatgct
 421 ataggaactt aactcttgtt tacatcaatc agcctagggt aaaaatggtt ttgttatatg
 481 ttgttttgcg taaagtcatg gtttccagga acttatggaa gacactgagg attactccaa
 541 tacttgtagt aaatttggga gaaaaaggca aggactaaag ttactactcc taacacaaat
 601 gagacggtca tggtgtgccc catcaaaaaa ggagagaaag agagacagac acatagaaac
 661 acgtgcacat acacacacac acacacacac acacacacac acacagagag agagagagag
 721 agagagagag agggagagag actatagaaa catgatgtta taaataacaa tagtgctgag
 781 gaagatgaat tagtgtaaaa ttgtattttt tgttctgagg ataggttaca tagatgattc
 841 taatccattt attattttta cattccaggc tacctaaaag aagacagtta tctcatattt
 901 ggctgccagc tttttatctt tctctcgacc acttaaaact tcagacttcc tgtcctgctg
 961 gtatcATGGA GAAAGTCCAA TACCTCACTC GCTCAGCTAT AAGAAGAGCC TCAACCATTG
1021 AAATGCCTCA ACAAGCACGT CAAAAGCTAC AGAATCTATT TATCAATTTC TGTCTCATCT
1081 TAATATGTCT CTTGCTGATC TGTATCATCG TGATGCTTCT CTGAagttct gctacaacct
1141 ctagatctgc agcttgccac atcagcttaa aatctgtcat cccatgcaga caggaaaaca
1201 atattgtata acagaccact tcctgagtag aagagtttct ttgtgaaaag gtcaagatta
1261 agactaaaac ttattgttac catatgtatt catctgttgg atcttgtaaa catgaaaagg
1321 gctttatttt caaaaattaa cttcaaaata agtgtataaa atgcaactgt tgatttcctc
1381 aacatggctc acaaatttct atcccaaatc ttttctgaag atgaagagtt tagttttaaa
1441 actgcactgc caacaagttc acttcatata taaagcatta tttttactct tttgaggtga
1501 atataattta tattacaatg gaaaagcttc tttaatacta agtatttttc aggtcttcac
1561 caagtatcaa agtaataaca caaatgaagt gtcattattc aaaatagtcc actgactcct
1621 cacatctgtt atcttattat aaagaactat ttgtagtaac tatcagaatc tacattctaa
1681 aacagaaatt gtatttttc tatgccacat taacatcttt taaagttgat gagaatcaag
1741 tatggaaaag taaggccata ctcttacata ataaaattcc ttttaagtaa tttttcaaa
1801 gaatcacaga attctagtac atgtaggtaa atcataaatc tgttctaaga catatgatca
1861 acagatgaga actggtggtt aatatgtgac agtgagatta gtcatatcac taatatacta
1921 acaacagaat ctaatcttca tttaaggcac tgtagtgaat tatctgagct agagttacct
1981 agcttaccat actatatctt tggaatcatg aaaccttaag acttcagaat gattttgcag
2041 gttgtcttcc attccagcct aacatccaat gcaggcaagg aaaataaaag atttccagtg
2101 acagaaaaat atattatctc aagtattttt taaaaatata tgaattctct ctccaaatat
2161 taactaatta ttagattata ttttgaaatg aacttgttgg cccatctatt acatctacag
2221 ctgacccttg aacatggggg ttaggggagc tgacaattcg tgggtccgca aaatcttaac
2281 tacctaatag cctactattg accataaacc ttactgataa cataaacagt aaattaacac
2341 atattttgcg tgttatatgt attatacact atattcctac aataaagtaa gctagagaaa
2401 atgttattta gaaaatcata agaaagagaa aatatattta ctattcatta aatggaagtg
2461 ggtcaacata aaagtcttca ttctcattgt cttcacattg agtaggcaga ggaggagaaa
2521 gatggggagg aagagaaggc gttggtcttg cagtcttgtc ttaggggtgt ggggagtggg
2581 ggaaagaata ttcatgtata agtggaccct tgcaattcaa gcccttgttg ttcaagggtc
2641 aactgtaata ggatatagct atttttcttc ctctatcaac caaatggtaa gcatctattt
```

2701 tgtagtccac tctactgagc taaattatag atccagctat gctatttata attattttct
2761 tgatgaataa attttcaatt tctcctctga ccatttcaga acatcttcca ataactcata
2821 aaacaactga agtaaaattg agtgctggaa aatatattca ccaaactttg gtaatttaag
2881 ttgactaaag tttaaaatta agtctaaaat agtttacacc tatactgcat aatccaacaa
2941 ttttaatttc agttgaagac atgttactaa tataactatt attaaaagag tagaggatgt
3001 gtaattaacc atatcttcta aaacatggtt actaaaagaa tatgtaacat caatattgac
3061 cttggtttct tacacaagtg ttgctaactc aatagtgaag gagacactat taaattttct
3121 gaacccatga gagatactag agatggggag tggaaagtgt ttggttcagg gatatctgaa
3181 gaacagaagg gcagagattt cttaagtgac gcctcatcta caagctggaa attcctaaaa
3241 acaagtagaa agcttataaa caacaggtga tacactcacc tcactggttt tagtaaatta
3301 ccaatacaga aagtatccct agtcttaaaa acaagtggaa aatttgaact gattagtcat
3361 attcctttga ttacactgtt tgttacaata tttttctcag taaacagaaa taactaattt
3421 ttttgttctt cattctttga tagaaattaa aatcttattc tgtgaggatt acagaatact
3481 ataactcaaa ttataaagta gaataaactc tttaaataat tattcttcat cataaagtgt
3541 aaagaataag atataagaaa acaatttatt tttaaaattt aatatactaa atgctcaaat
3601 atgttctact atagaataag ttcttatctt aatttacagg gcactaaaaa caattttaaa
3661 atgcttaatg ttgcctttta tattttaatt ggttaagaat atatatttgt ttaatgcaaa
3721 tcagaatcac tatattaaaa tgaatgttct tgaaaactca gtggggctgc tctataatac
3781 acttacaatt gatactaacg ataaaacgct ctaattatag ttttaatttt ataggggttt
3841 ttttggtgac tttccccaaa taaaacaaaa tcaagacgcc caagtccaga cacacaagag
3901 ggcagacgta tataagataa caaattgcta ctacatagcg attatttatc ttttgattat
3961 ttttgagact tctggcaaag taggactata ataaattttc agaaaagatc tgctcttctg
4021 ctaagtaaca gcacacttca aaatgtccag aaattttatg ctaatattac atagaaaact
4081 tgacagatga aaaaagtgaa acacattact aattttattc attaaaacag gtatataaaa
4141 gtcctcttta actacctaat gaatgtgtca tatcctaata tagggagtgt catagtgttc
4201 acatctttga atatataaat tatctcaaac agttaaaaaa ttaaagccag aacagggtac
4261 actagcttgg ctgcaggtcg tcgaaattct accgggtagg ggaggcgctt ttcccaaggc
4321 agtctggagc atgcgcttta gcagccccgc tgggcacttg gcgctacaca agtggcctct
4381 ggcctcgcac acattccaca tccaccggta ggcgccaacc ggctccgttc tttggtggcc
4441 ccttcgcgcc accttctact cctcccctag tcaggaagtt cccccccgcc ccgcagctcg
4501 cgtcgtgcag gacgtgacaa atggaagtag cacgtctcac tagtctcgtg cagatggaca
4561 gcaccgctga gcaatggaag cgggtaggcc tttggggcag cggccaatag cagctttgct
4621 ccttcgcttt ctgggctcag aggctgggaa ggggtgggtc cgggggcggg ctcaggggcg
4681 ggctcagggg cggggcgggc gcccgaaggt cctccggagg cccggcattc tgcacgcttc
4741 aaaagcgcac gtctgccgcg ctgttctcct cttcctcatc tccgggcctt tcgacctgca
4801 gcctgttgac aattaatcat cggcatagta tatcggcata gtataatacg acaaggtgag
4861 gaactaaacc atgggatcgg ccattgaaca agatggattg cacgcaggtt ctccggccgc
4921 ttgggtggag aggctattcg gctatgactg ggcacaacag acaatcggct gctctgatgc
4981 cgccgtgttc cggctgtcag cgcaggggcg cccggttctt tttgtcaaga ccgacctgtc
5041 cggtgccctg aatgaactgc aggacgaggc agcgcggcta tcgtggctgg ccacgacggg
5101 cgttccttgc gcagctgtgc tcgacgttgt cactgaagcg ggaagggact ggctgctatt
5161 gggcgaagtg ccggggcagg atctcctgtc atctcacctt gctcctgccg agaaagtatc
5221 catcatggct gatgcaatgc ggcggctgca tacgcttgat ccggctacct gcccattcga
5281 ccaccaagcg aaacatcgca tcgagcgagc acgtactcgg atggaagccg gtcttgtcga
5341 tcaggatgat ctggacgaag agcatcaggg gctcgcgcca gccgaactgt tcgccaggct
5401 caaggcgcgc atgcccgacg gcgatgatct cgtcgtgacc catggcgatg cctgcttgcc
5461 gaatatcatg gtggaaaatg gccgcttttc tggattcatc gactgtggcc ggctgggtgt
5521 ggcggaccgc tatcaggaca tagcgttggc tacccgtgat attgctgaag agcttggcgg
5581 cgaatgggct gaccgcttcc tcgtgcttta cggtatcgcc gctcccgatt cgcagcgcat
5641 cgccttctat cgccttcttg acgagttctt ctgaggggat caattctcta gagctcgctg
5701 atcagcctcg actgtgcctt ctagttgcca gccatctgtt gtttgcccct cccccgtgcc
5761 ttccttgacc ctggaaggtg ccactcccac tgtcctttcc taataaaatg aggaaattgc
5821 atcgcattgt ctgagtaggt gtcattctat tctggggggt ggggtggggc aggacagcaa
5881 gggggaggat tgggaagaca atagcaggca tgctggggat gcggtgggct ctatggcttc
5941 tgaggcggaa agaaccagct ggggctcgac tagagcttgc ggaaccctta atATAACTTC
6001 GTATAATGTA TGCTATACGA AGTTATggat ccttggggtc tttattgaga agtttggtga
6061 tgtttttgtg accagaaata tgctatagga acttaactct tgtttacatc aatcagccta
6121 gggtaaaaat ggttttgtta tatgttgttt tgcgtaaagt catggtttcc aggaacttat
6181 ggaagacact gaggattact ccaatacttg tagtaaattt gggagaaaaa ggcaaggact
6241 aaagttacta ctcctaacac aaatgagacg gtcatggtgt gccccatcaa aaaaggagag
6301 aaagagagac agacacatag aaacacgtgc acatacacac acacacacac acacacacac

6361 acacacacag agagagagag agagagagag agagagggag agagactata gaaacatgat
6421 gttataaata acaatagtgc tgaggaagat gaattagtgt aaaattgtat tttttgttct
6481 gaggataggt tacatagatg attctaatcc atttattatt tttacattcc aggctaccta
6541 aaagaagaca gttatctcat atttggctgc cagcttttta tctttctctc gaccacttaa
6601 aacttcagac ttcctgtcct gctggtatcA TGGAGAAAGT CCAATACCTC ACTCGCTCAG
6661 CTATAAGA-- -GCCTCAACC ATTGAAATGC CTCAACAAGC ACGTCAAAAG CTACAGAATC
6721 TATTTATCAA TTTCTGTCTC ATCTTAATAT GTCTCTTGCT GATCTGTATC ATCGTGATGC
6781 TTCTCTGAag ttctgctaca acctctagat <u>ctgcag</u>cttg ccacatcagc ttaaaatctg
6841 tcatcccatg cagacaggaa aacaatattg tataacagac cacttcctga gtagaagagt
6901 ttctttgtga aaaggtcaag attaagacta aaacttattg ttaccatatg tattcatctg
6961 ttggatcttg taaacatgaa aagggcttta ttttcaaaaa ttaacttcaa aataagtgta
7021 taaaatgcaa ctgttgattt cctcaacatg gctcacaaat ttctatccca aatcttttct
7081 gaagatgaag agtttagttt taaaactgca ctgccaacaa gttcacttca tatataaagc
7141 attattttta ctcttttgag gtgaatataa tttatattac aatggaaaag cttctttaat
7201 actaagtatt tttcaggtct tcaccaagta tcaaagtaat aacacaaatg aagtgtcatt
7261 attcaaaata gtccactgac tcctcacatc tgttatctta ttataaagaa ctatttgtag
7321 taactatcag aatctacatt ctaaaacaga aattgtattt tttctatgcc acattaacat
7381 cttttaaagt tgatgagaat caagtatgga aaagtaaggc catactctta cataataaaa
7441 ttccttttaa gtaatttttt caaagaatca cagaattcta gtacatgtag gtaaatcata
7501 aatctgttct aagacatatg atcaacagat gagaactggt ggttaatatg tgacagtgag
7561 attagtcata tcactaatat actaacaaca gaatctaatc ttcatttaag gcactgtagt
7621 gaattatctg agctagagtt acctagctta ccatactata tctttggaat catgaaacct
7681 taagacttca gaatgatttt gcaggttgtc ttccattcca gcctaacatc caatgcaggc
7741 aaggaaaata aaagatttcc agtgacagaa aaatatatta tctcaagtat tttttaaaaa
7801 tatatgaatt ctctctccaa atattaacta attattagat tatattttga aatgaacttg
7861 ttggcccatc tattacatct acagctgacc cttgaacatg gggttaggg gagctgacaa
7921 ttcgtgggtc cgcaaaatct taactaccta atagcctact attgaccata aaccttactg
7981 ataacataaa cagtaaatta acacatattt tgcgtgttat atgtattata cactatattc
8041 ctacaataaa gtaagctaga gaaaatgtta tttagaaaat cataagaaag agaaaatata
8101 tttactattc attaaatgga agtgggtcaa cataaaagtc ttcattctca ttgtcttcac
8161 attgagtagg cagaggagga gaaagatggg gaggaagaga aggcgttggt cttgcagtct
8221 tgtcttaggg gtgtggggag tgggggaaag aatattcatg tataagtgga cccttgcaat
8281 tcaagccctt gttgttcaag ggtcaactgt aataggatat agctattttt cttcctctat
8341 caaccaaatg gtaagcatct attttgtagt ccactctact gagctaaatt atagatccag
8401 ctatgctatt tataattatt ttcttgatga ataaattttc aatttctcct ctgaccattt
8461 cagaacatct tccaataact cataaaacaa ctgaagtaaa attgagtgct ggaaaatata
8521 ttcaccaaac tttggtaatt taagttgact aaagtttaaa attaagtcta aaatagttta
8581 cacctatact gcataatcca acaattttaa tttcagttga agacatgtta ctaatataac
8641 tattattaaa agagtagagg atgtgtaatt aaccatatct tctaaaacat ggttactaaa
8701 agaatatgta acatcaatat tgaccttggt ttcttacaca agtgttgcta actcaatagt
8761 gaaggagaca ctattaaatt ttctgaaccc atgagagata ctagagatgg ggagtggaaa
8821 gtgtttggtt cagggatatc tgaagaacag aagggcagag atttcttaag tgacgcctca
8881 tctacaagct ggaaattcct aaaaacaagt agaaagctta taaacaacag gtgatacact
8941 cacctcactg gttttagtaa attaccaata cagaaagtat ccctagtctt aaaaacaagt
9001 ggaaaatttg aactgattag tcatattcct ttgattacac tgtttgttac aatatttttc
9061 tcagtaaaca gaaataacta atttttttgt tcttcattct ttgatagaaa ttaaaatctt
9121 attctgtgag gattacagaa tactataact caaattataa agtagaataa actctttaaa
9181 taattattct tcatcataaa gtgtaaagaa taagatataa gaaaacaatt tatttttaaa
9241 atttaatata ctaaatgctc aaatatgttc tactatagaa taagttctta tcttaattta
9301 cagggcacta aaaacaattt taaaatgctt aatgttgcct tttatatttt aattggttaa
9361 gaatatatat ttgtttaatg caaatcagaa tcactatatt aaaatgaatg ttcttgaaaa
9421 ctcagtgggg ctgctctata atacacttac aattgatact aacgataaaa cgctctaatt
9481 atagttttaa ttttataggg gtttttttgg tgactttccc caaataaaac aaaatcaaga
9541 cgcccaagtc cagacacaca agagggcaga cgtatataag ataacaaatt gctactacat
9601 agcgattatt tatcttttga ttatttttga gacttctggc aaagtaggac tataataaat
9661 tttcagaaaa gatctgctct tctgctaagt aacagcacac ttcaaaatgt ccagaaattt
9721 tatgctaata ttacatagaa aacttgacag atgaaaaaag tgaaacacat tactaatttt
9781 attcattaaa acaggtatat aaaagtcctc tttaactacc taatgaatgt gtcatatcct
9841 aatataggga gtgtcatagt gttcacatct ttgaatatat aaattatctc aaacagttaa
9901 aaaattaaag ccagaacagg gtac<u>actagt</u> aaaaataaaa ataagaaaac tttcttttca
9961 gaggatgtag tagtatacat aaactaccct cacattaaaa atagaacctt ttcaaattga

```
10021 aactacaatg aggtaccaac tcacaccagt cagaatggcc atcattagta aatccacaaa
10081 aaataaatgc tgaagagggg gtggagaaaa gggaaccctc ctaaactgct ggtgggaatg
10141 taaattggta taactactat ggaaagaagt atggaggttc ctcagaaaat taaatataga
10201 actaccctat gatccagcaa tcccactcct
```

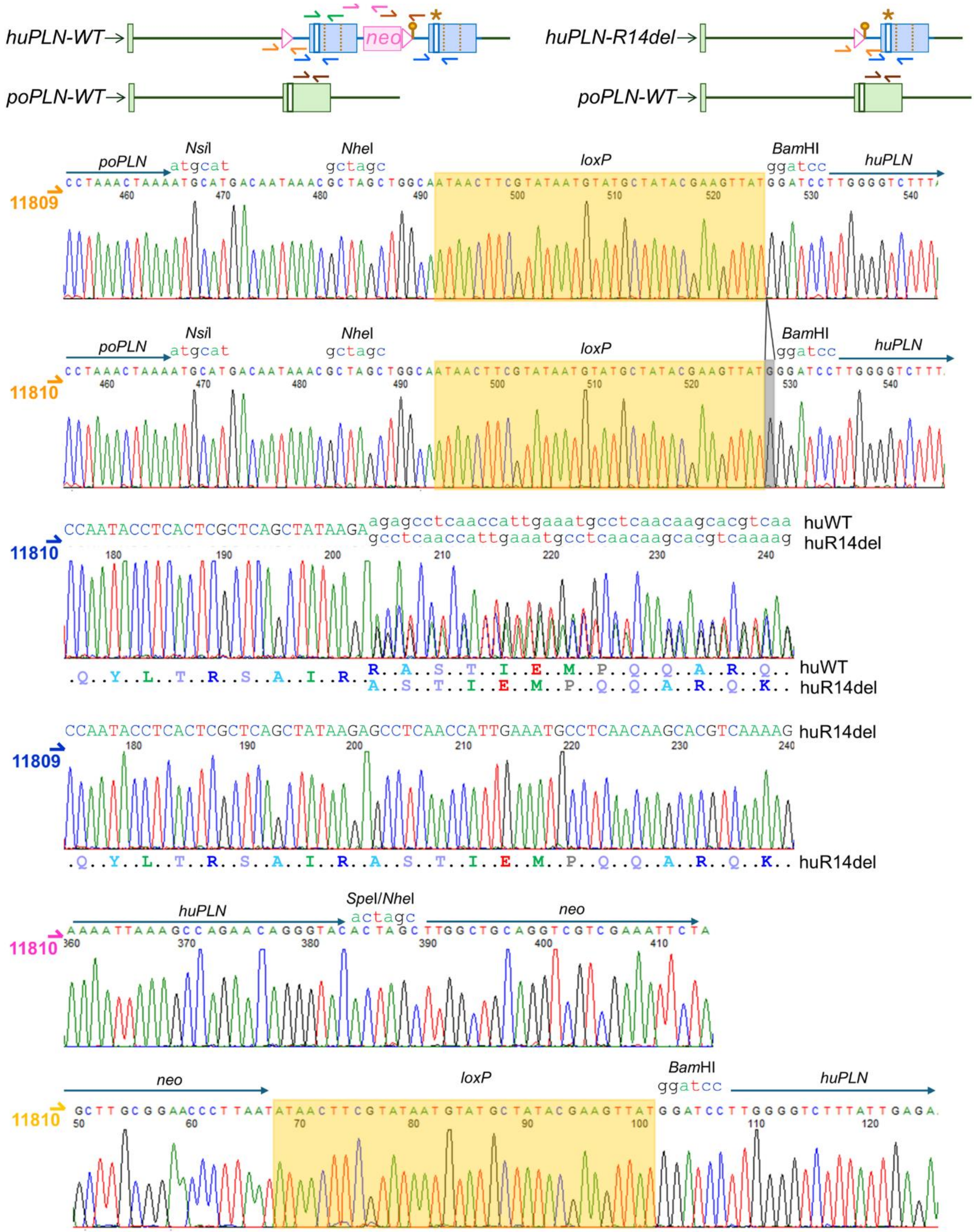

Fig. S1. Sequence details of PLN founder animals. Localization and specificity of PCR products on genomic DNA in huPLN-WT/poPLN-WT and huPLN-R14del/poPLN-WT founder animals are indicated (top panel). Representative Sanger electropherograms are shown. Orange: spanning first lox site, indicating the appearance of a 2xG motif in huPLN-R14del (#11809) vs 3xG in huPLN-WT (#11810). Blue: spanning open reading frame, indicating that huPLN-WT #11810 comprise both cassettes (mixed pattern with R14 codon), whereas huPLN-R14del retains only the downstream cassette.

Amino acid sequences are indicated in one-letter code. Transition from huPLN-WT to neo cassette (pink) and transition from neo cassette to huPLN-R14del cassette (yellow) only appear in huPLN-WT animal #11810.
