# Supplementary material for: Activating a patient-relevant pathogenic variant of a genetically defined heart disease in a humanized pig model
Source: Lab Anim (NY). 2026 Sep 11;55(10):386–401. doi: 10.1038/s41684-026-01787-6 (PMC13619452; doi:10.1038/s41684-026-01787-6)
Supplement: Supplementary file 2 [file 41684_2026_1787_MOESM2_ESM.pdf]

nature portfolio

Corresponding author(s): Nikolai Klymiuk

Last updated by author(s): Feb 9, 2026

Reporting Summary

Nature Portfolio wishes to improve the reproducibility of the work that we publish. This form provides structure for consistency and transparency in reporting. For further information on Nature Portfolio policies, see our Editorial Policies and the Editorial Policy Checklist.

Statistics

For all statistical analyses, confirm that the following items are present in the figure legend, table legend, main text, or Methods section.

n/a	Confirmed	
☐	☒	The exact sample size (*n*) for each experimental group/condition, given as a discrete number and unit of measurement
☐	☒	A statement on whether measurements were taken from distinct samples or whether the same sample was measured repeatedly
☐	☒	The statistical test(s) used AND whether they are one- or two-sided<br>*Only common tests should be described solely by name; describe more complex techniques in the Methods section.*
☒	☐	A description of all covariates tested
☒	☐	A description of any assumptions or corrections, such as tests of normality and adjustment for multiple comparisons
☐	☐	A full description of the statistical parameters including central tendency (e.g. means) or other basic estimates (e.g. regression coefficient) AND variation (e.g. standard deviation) or associated estimates of uncertainty (e.g. confidence intervals)
☒	☐	For null hypothesis testing, the test statistic (e.g. *F*, *t*, *r*) with confidence intervals, effect sizes, degrees of freedom and *P* value noted<br>*Give P values as exact values whenever suitable.*
☒	☐	For Bayesian analysis, information on the choice of priors and Markov chain Monte Carlo settings
☒	☐	For hierarchical and complex designs, identification of the appropriate level for tests and full reporting of outcomes
☒	☐	Estimates of effect sizes (e.g. Cohen's *d*, Pearson's *r*), indicating how they were calculated

Our web collection on statistics for biologists contains articles on many of the points above.

Software and code

Policy information about availability of computer code

Data collection	not applicable, no bio-informatics work conducted
Data analysis	not applicable, no bio-informatics work conducted

For manuscripts utilizing custom algorithms or software that are central to the research but not yet described in published literature, software must be made available to editors and reviewers. We strongly encourage code deposition in a community repository (e.g. GitHub). See the Nature Portfolio guidelines for submitting code & software for further information.

Data

Policy information about availability of data

All manuscripts must include a data availability statement. This statement should provide the following information, where applicable:

- Accession codes, unique identifiers, or web links for publicly available datasets
- A description of any restrictions on data availability
- For clinical datasets or third party data, please ensure that the statement adheres to our policy

yes, data are provided upon request

Research involving human participants, their data, or biological material

Policy information about studies with human participants or human data. See also policy information about sex, gender (identity/presentation), and sexual orientation and race, ethnicity and racism.

Reporting on sex and gender	PLN-R14del carriers of both sexes were explored for SNV in the PLN gene. All showed consistent pattern
Reporting on race, ethnicity, or other socially relevant groupings	PLN carriers descend from a single founder event in the medieval ages, resulting in a predominantly North-Holland background of the PLN carrier population.
Population characteristics	PLN carriers descend from a single founder event in the medieval ages, resulting in a predominantly North-Holland background of the PLN carrier population.
Recruitment	Diseased carriers identifed upon clinical visits at UMC Utrecht. Related familiy members recruited by the PLN foundation
Ethics oversight	under supervision of UMC Utrecht ethics committee

Note that full information on the approval of the study protocol must also be provided in the manuscript.

Field-specific reporting

Please select the one below that is the best fit for your research. If you are not sure, read the appropriate sections before making your selection.

☒ Life sciences ☐ Behavioural & social sciences ☐ Ecological, evolutionary & environmental sciences

For a reference copy of the document with all sections, see nature.com/documents/nr-reporting-summary-flat.pdf

Life sciences study design

All studies must disclose on these points even when the disclosure is negative.

Sample size	indicated,
Data exclusions	no data excluded
Replication	instead of technical replicates, we performed complementary analysis methods
Randomization	small data sets, small experimental cohorts, not performed
Blinding	not conducted due to small cohorts

Behavioural & social sciences study design

All studies must disclose on these points even when the disclosure is negative.

Study description	not applicable, no behavioral & social science studies conducted
Research sample	not applicable, no behavioral & social science studies conducted
Sampling strategy	not applicable, no behavioral & social science studies conducted
Data collection	not applicable, no behavioral & social science studies conducted
Timing	not applicable, no behavioral & social science studies conducted
Data exclusions	not applicable, no behavioral & social science studies conducted
Non-participation	not applicable, no behavioral & social science studies conducted
Randomization	not applicable, no behavioral & social science studies conducted

Ecological, evolutionary & environmental sciences study design

All studies must disclose on these points even when the disclosure is negative.

Study description	not applicable, no experimental ecological evolutionary and environmental studies conducted
Research sample	not applicable, no experimental ecological evolutionary and environmental studies conducted
Sampling strategy	not applicable, no experimental ecological evolutionary and environmental studies conducted
Data collection	not applicable, no experimental ecological evolutionary and environmental studies conducted
Timing and spatial scale	not applicable, no experimental ecological evolutionary and environmental studies conducted
Data exclusions	not applicable, no experimental ecological evolutionary and environmental studies conducted
Reproducibility	not applicable, no experimental ecological evolutionary and environmental studies conducted
Randomization	not applicable, no experimental ecological evolutionary and environmental studies conducted
Blinding	not applicable, no experimental ecological evolutionary and environmental studies conducted

Did the study involve field work? ☐ Yes ☒ No

Field work, collection and transport

Field conditions	not applicable
Location	not applicable
Access & import/export	not applicable
Disturbance	not applicable

Reporting for specific materials, systems and methods

We require information from authors about some types of materials, experimental systems and methods used in many studies. Here, indicate whether each material, system or method listed is relevant to your study. If you are not sure if a list item applies to your research, read the appropriate section before selecting a response.

Materials & experimental systems

n/a	Involved in the study	
☒	☐	Antibodies
☐	☒	Eukaryotic cell lines
☒	☐	Palaeontology and archaeology
☐	☒	Animals and other organisms
☒	☐	Clinical data
☒	☐	Dual use research of concern
☒	☐	Plants

Methods

n/a	Involved in the study	
☒	☐	ChIP-seq
☒	☐	Flow cytometry
☒	☐	MRI-based neuroimaging

Antibodies

Antibodies used	no
Validation	not applicable

Eukaryotic cell lines

Policy information about cell lines and Sex and Gender in Research

Cell line source(s)	Primary kidney cells
Authentication	pig origin
Mycoplasma contamination	not determined
Commonly misidentified lines (See ICLAC register)	primary cell line, in house cultivated

Palaeontology and Archaeology

Specimen provenance	not applicable, no palaentology and archeology study conducted
Specimen deposition	not applicable, no palaentology and archeology study conducted
Dating methods	not applicable, no palaentology and archeology study conducted

☐ Tick this box to confirm that the raw and calibrated dates are available in the paper or in Supplementary Information.

Ethics oversight	not applicable

Note that full information on the approval of the study protocol must also be provided in the manuscript.

Animals and other research organisms

Policy information about studies involving animals; ARRIVE guidelines recommended for reporting animal research, and Sex and Gender in Research

Laboratory animals	pig
Wild animals	no
Reporting on sex	yes, PKC population of female sex, resulting in SCNT-derived founder pigs. male and female animals produced by breeding from this founder pig.
Field-collected samples	no
Ethics oversight	yes, approval numbers of animal ethics indicated

Note that full information on the approval of the study protocol must also be provided in the manuscript.

Clinical data

Policy information about clinical studies

All manuscripts should comply with the ICMJE guidelines for publication of clinical research and a completed CONSORT checklist must be included with all submissions.

Clinical trial registration	no clinical data included
Study protocol	not applicable
Data collection	not applicable
Outcomes	not applicable

Dual use research of concern

Policy information about dual use research of concern

Hazards

Could the accidental, deliberate or reckless misuse of agents or technologies generated in the work, or the application of information presented in the manuscript, pose a threat to:

No	Yes	
☒	☐	Public health
☒	☐	National security
☒	☐	Crops and/or livestock
☒	☐	Ecosystems
☒	☐	Any other significant area

Experiments of concern

Does the work involve any of these experiments of concern:

No	Yes	
☒	☐	Demonstrate how to render a vaccine ineffective
☒	☐	Confer resistance to therapeutically useful antibiotics or antiviral agents
☒	☐	Enhance the virulence of a pathogen or render a nonpathogen virulent
☒	☐	Increase transmissibility of a pathogen
☒	☐	Alter the host range of a pathogen
☒	☐	Enable evasion of diagnostic/detection modalities
☒	☐	Enable the weaponization of a biological agent or toxin
☒	☐	Any other potentially harmful combination of experiments and agents

Plants

Seed stocks	no plant work included in this study
Novel plant genotypes	not applicable
Authentication	not applicable

ChIP-seq

Data deposition

☐ Confirm that both raw and final processed data have been deposited in a public database such as GEO.

☐ Confirm that you have deposited or provided access to graph files (e.g. BED files) for the called peaks.

Data access links<br>*May remain private before publication.*	not applicable, no ChIP seq studies conducted
Files in database submission	not applicable, no ChIP seq studies conducted
Genome browser session<br>(e.g. UCSC)	not applicable, no ChIP seq studies conducted

Methodology

Replicates	not applicable
Sequencing depth	not applicable
Antibodies	not applicable
Peak calling parameters	not applicable
Data quality	not applicable
Software	not applicable

Flow Cytometry

Plots

Confirm that:

☒ The axis labels state the marker and fluorochrome used (e.g. CD4-FITC).

☒ The axis scales are clearly visible. Include numbers along axes only for bottom left plot of group (a 'group' is an analysis of identical markers).

☒ All plots are contour plots with outliers or pseudocolor plots.

☒ A numerical value for number of cells or percentage (with statistics) is provided.

Methodology

Sample preparation	primary cells fixed in 70% EtOH and then permeabilized and stained with DAPI
Instrument	Beckman Coulter (488 nm excitation and 530/30 nm bandpass emission filter for GFP laser) and LSRFortessa Cell Analyzer (405 nm excitation and 450/50 nm bandpass emission filter for DAPI, and 488 nm excitation and 530/30 nm bandpass emission filter for GFP)
Software	Flow Jo
Cell population abundance	not applicable
Gating strategy	indicated in Fig. 6f & Fig. 6g

☐ Tick this box to confirm that a figure exemplifying the gating strategy is provided in the Supplementary Information.

Magnetic resonance imaging

Experimental design

Design type	no MRI experiment studies conducted
Design specifications	no MRI experiment studies conducted
Behavioral performance measures	no MRI experiment studies conducted

Acquisition

Imaging type(s)	not applicable
Field strength	not applicable
Sequence & imaging parameters	not applicable
Area of acquisition	not applicable

Diffusion MRI ☐ Used ☒ Not used

Preprocessing

Preprocessing software	not applicable
Normalization	not applicable
Normalization template	not applicable
Noise and artifact removal	not applicable
Volume censoring	not applicable

Statistical modeling & inference

Model type and settings	not applicable
Effect(s) tested	not applicable

Specify type of analysis: ☐ Whole brain ☐ ROI-based ☐ Both

Statistic type for inference<br>(See Eklund et al. 2016)	not applicable
Correction	not applicable

Models & analysis

n/a	Involved in the study
☒	☐ Functional and/or effective connectivity
☒	☐ Graph analysis
☐	☐ Multivariate modeling or predictive analysis

Functional and/or effective connectivity	not applicable
Graph analysis	not applicable
Multivariate modeling and predictive analysis	not applicable
