# Supplementary material for: Activating a patient-relevant pathogenic variant of a genetically defined heart disease in a humanized pig model
Source: Lab Anim (NY). 2026 Sep 11;55(10):386–401. doi: 10.1038/s41684-026-01787-6 (PMC13619452; doi:10.1038/s41684-026-01787-6)
Supplement: Supplementary file 3 [file 41684_2026_1787_MOESM3_ESM.pdf]

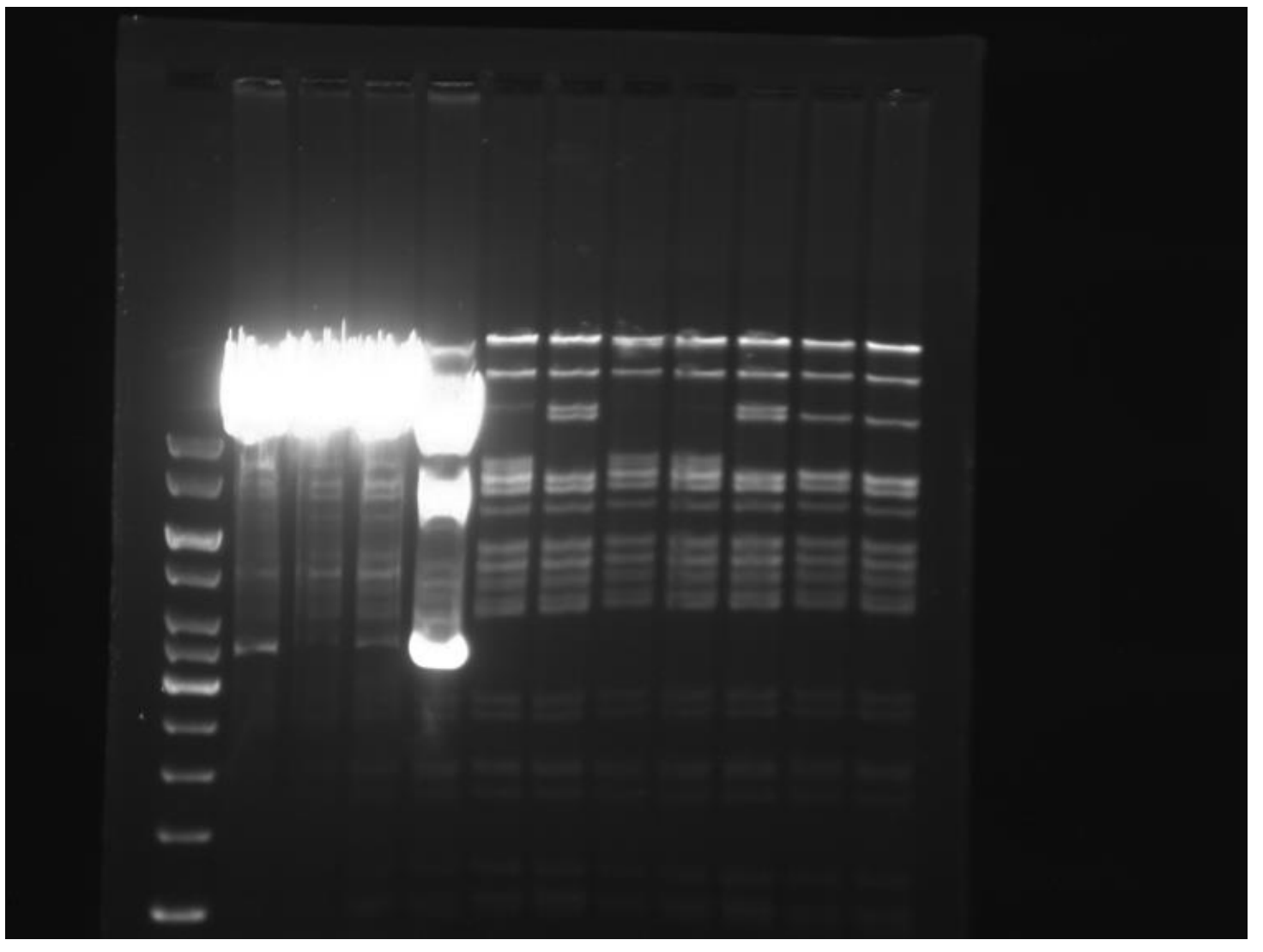

Fig. 4b
BAC RE fingerprinting
Enzyme: NcoI
Sample order (from left to right):
Marker (Thermo 1kb ladder)
5 lanes of incorrect clones
B22 (modified BAC clone)
B23 (modified BAC clone)
B24 (modified BAC clone)
B25 (modified BAC clone)
Original CH242-318M5
Original CH242-318M5
