# Supplementary material for: Activating a patient-relevant pathogenic variant of a genetically defined heart disease in a humanized pig model
Source: Lab Anim (NY). 2026 Sep 11;55(10):386–401. doi: 10.1038/s41684-026-01787-6 (PMC13619452; doi:10.1038/s41684-026-01787-6)

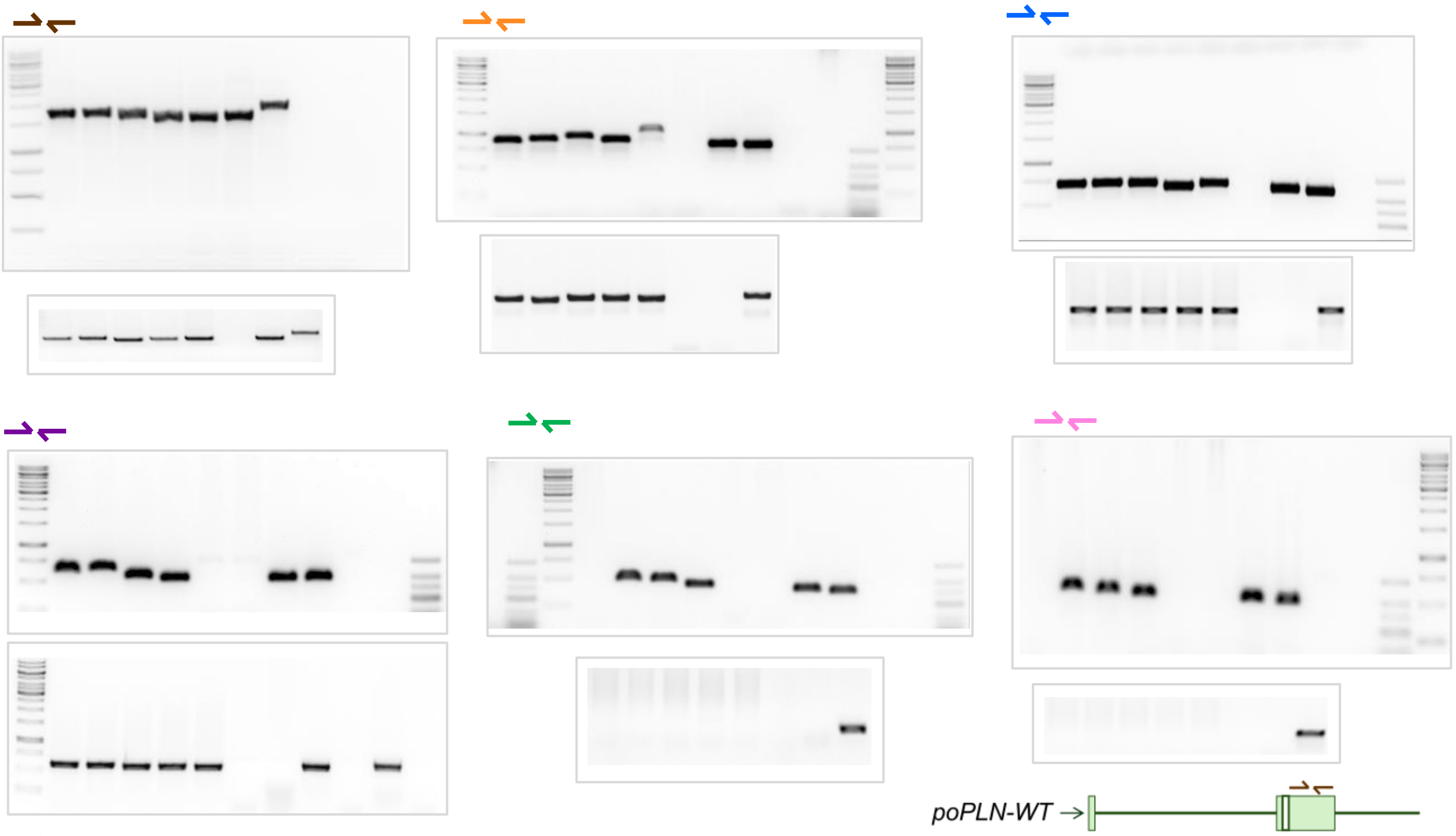

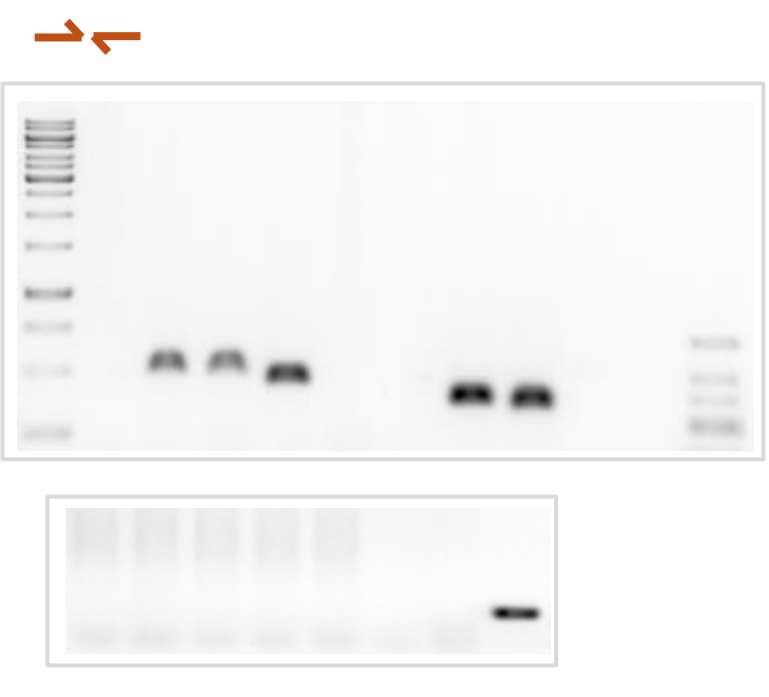

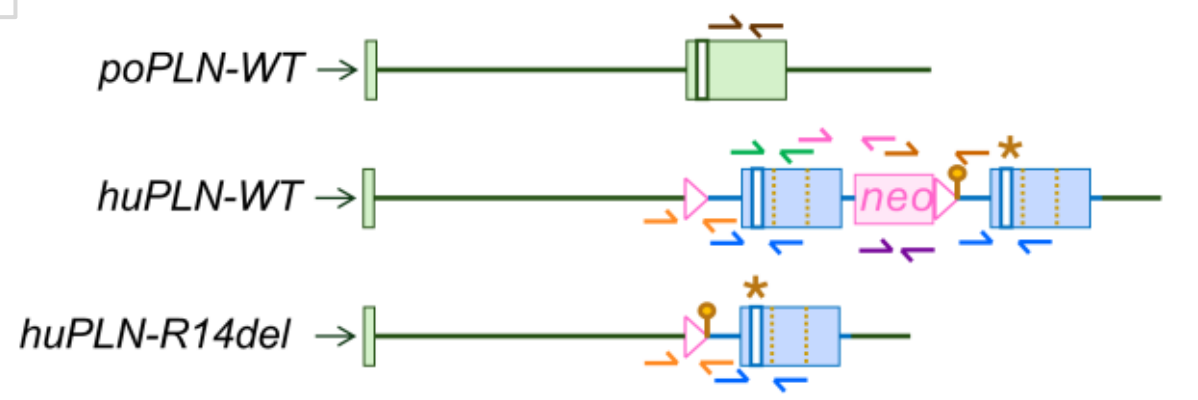

Fig. 7c

Genotyping of cloned & re-cloned offspring genotyping :

<u>Upper panels, cloned founders:</u> Marker - #11809, #11820, #11926, #11927, #11928, WT-gDNA, WT-gDNA + mod. BAC, modBAC, NTC

<u>Lower panel recloned founders:</u> Marker,#12435, #12436, #12437, #12438, #12439, #12440, WT-gDNA, WT-gDNA + mod. BAC

Fig. 7i

Genotyping of F1 piglets, from 11810 x 11809 inbreeding

Left gels: Thermo 1kb ladder - #62 - #63 - #64 - #65 - #66 - #67 - #68 - #69 - #70 - #71 - #72

Right gels: Thermo 1kb ladder - #73 - #74 - #75 - #76 - #77 - poWT ctrl - huWT ctrl - huR14del ctrl

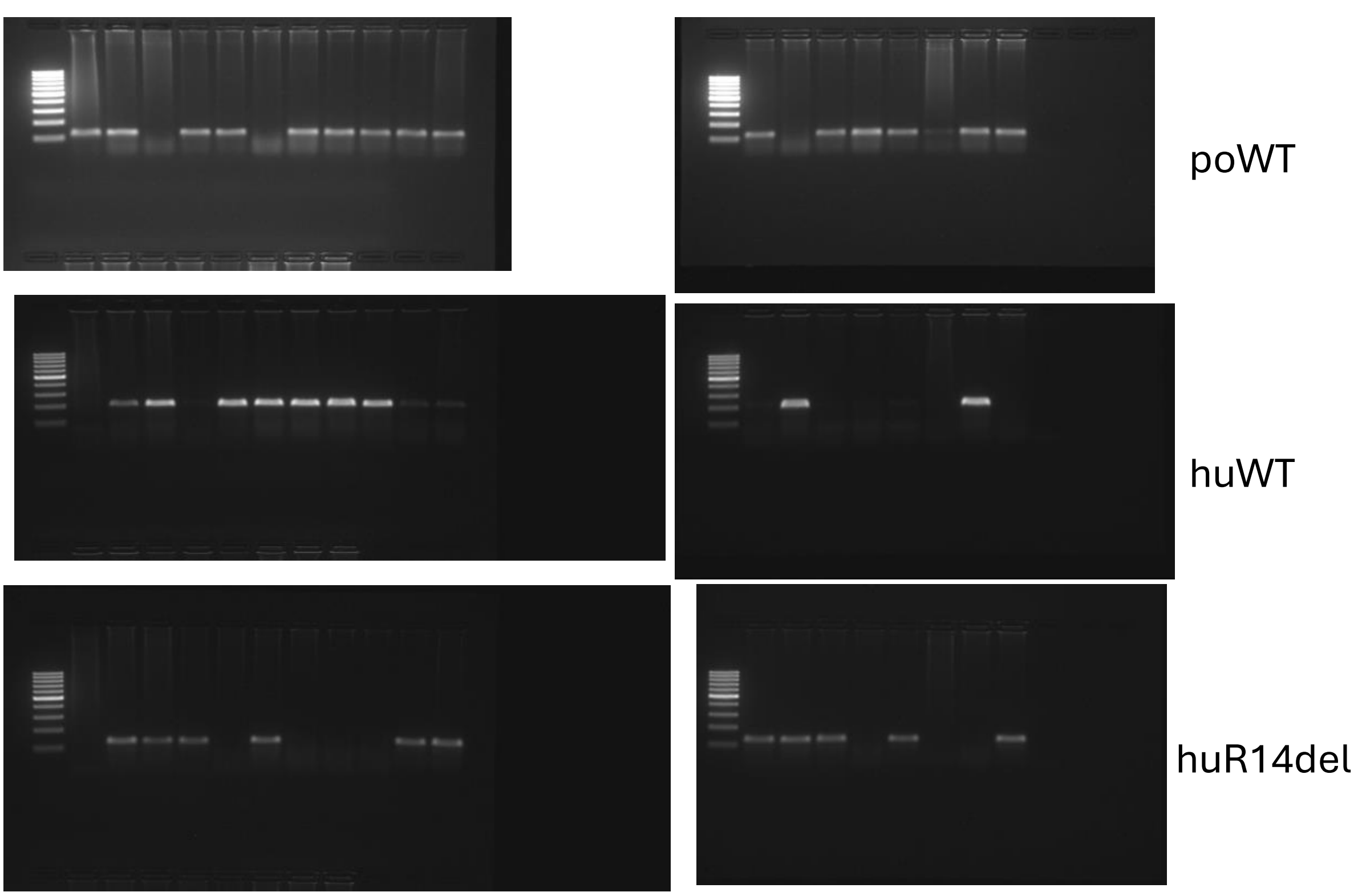

Supplement: Supplementary file 4 [file 41684_2026_1787_MOESM4_ESM.pdf]
