# Supplementary material for: Activating a patient-relevant pathogenic variant of a genetically defined heart disease in a humanized pig model
Source: Lab Anim (NY). 2026 Sep 11;55(10):386–401. doi: 10.1038/s41684-026-01787-6 (PMC13619452; doi:10.1038/s41684-026-01787-6)

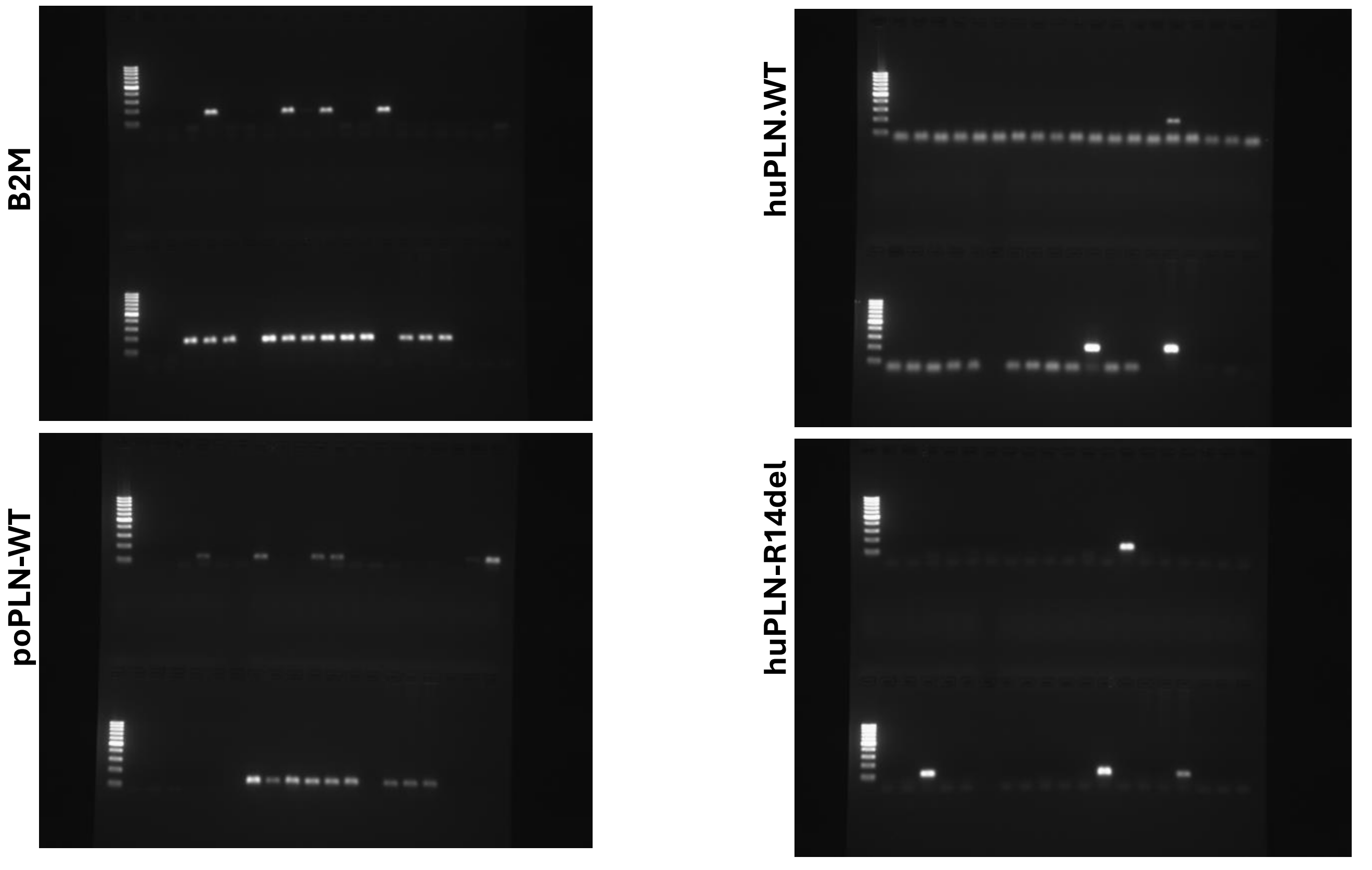

Fig. 9a. Genotyping of Cre-injected embryos derived from huPLN-WT x poWT matings
Sample arrangement on gels:
Upper line: Marker – em1 – em2 – em3 – em4 – em5 – em6 – em7 – em 8 – em9 – em10 – em11 – em12 – em13 – em14 – em15 – em16 – em17 – em18 – em19
Lower line: Marker – em20 – em21 – em22 – em24 – empty – 3x parthenote-derived blastocysts – blastocysts spiked with 10 copies of genomic DNA without PLN genetic modification – blastocysts spiked with 10 copies genomic DNA with huPLN-WT genotype – blastocysts spiked with 10 copies genomic DNA with huPLN-R14del genotype – non-template control – blastocysts spiked with 2500 copies of poPLN-WT, huPLN-WT and huPLN-R14del genotypes – non-template control

Fig. 9b. Genotyping of piglets derived from Cre-injected embryos
Sample arrangement on gels:
Marker – DNA from skin of MP1, MP2, MP3, MP4, MP5 – DNA from blood of MP1, MP2, MP3, MP4, MP5 – controls of poPLN-WT, huPLN-WT, huPLN-R14del, non-template control

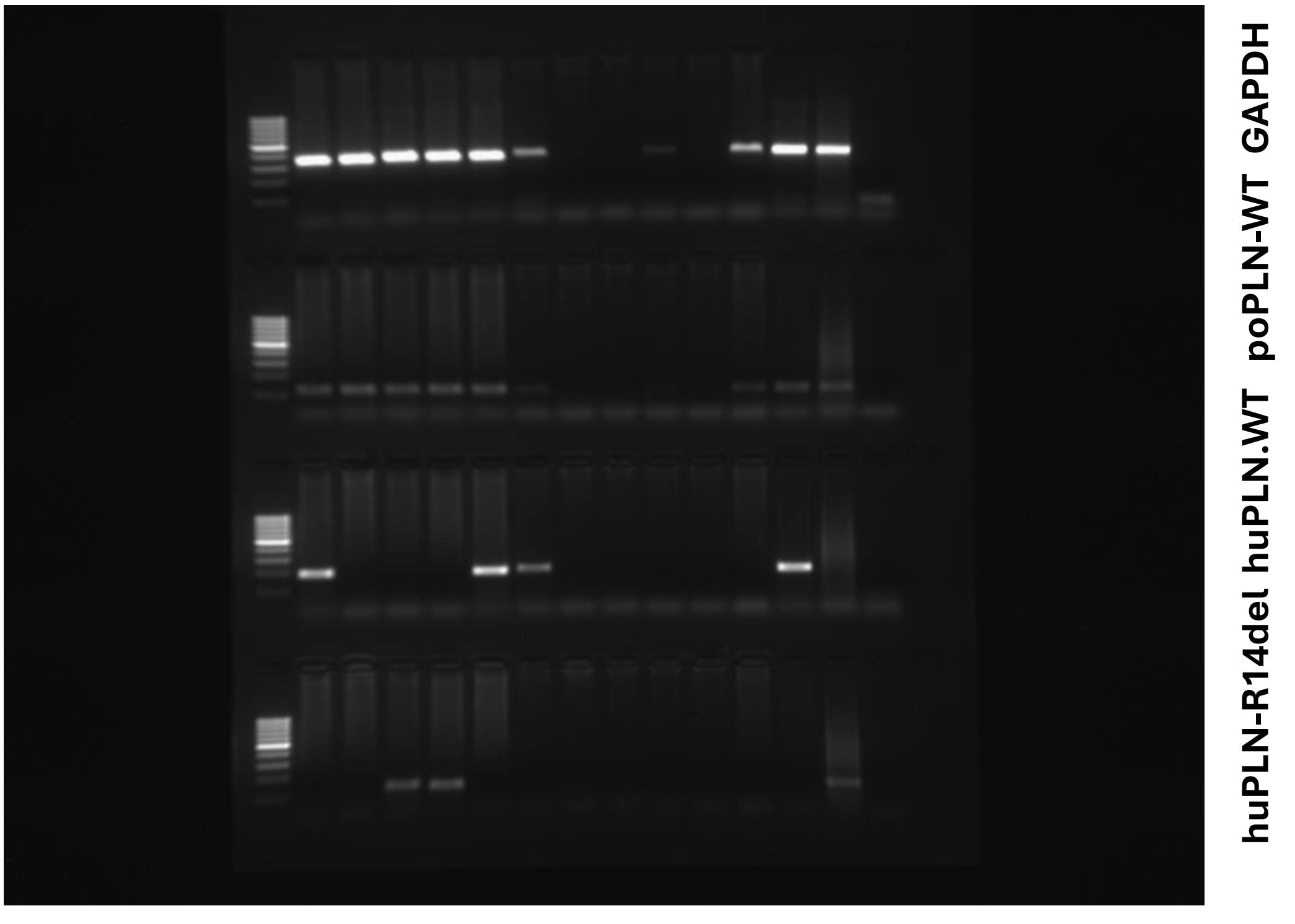

Supplement: Supplementary file 5 [file 41684_2026_1787_MOESM5_ESM.pdf]
