# Supplementary material for: Activating a patient-relevant pathogenic variant of a genetically defined heart disease in a humanized pig model
Source: Lab Anim (NY). 2026 Sep 11;55(10):386–401. doi: 10.1038/s41684-026-01787-6 (PMC13619452; doi:10.1038/s41684-026-01787-6)
Supplement: Supplementary file 6 [file 41684_2026_1787_MOESM6_ESM.pdf]

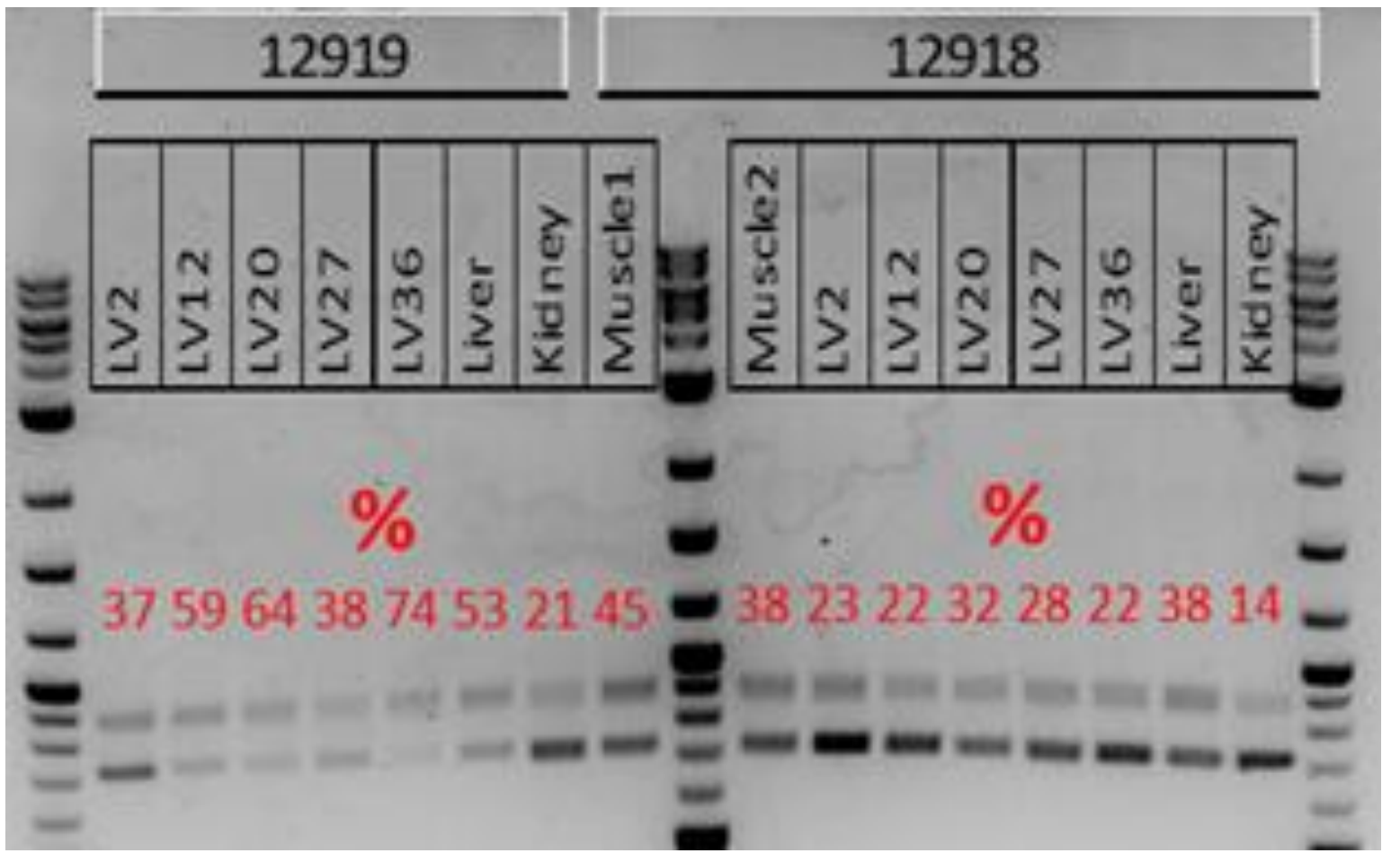

Fig. 10e
Restriction enzyme-induced fragment length polymorphism to determine post-natally activated R14del muations by Cre-AAV
An *Sap*I site in huPLN-WT but not huPLN-R14del discriminates both stages
